# Genomic Evolution of Breast Cancer Metastasis and Relapse

**DOI:** 10.1016/j.ccell.2017.07.005

**Published:** 2017-08-14

**Authors:** Lucy R. Yates, Stian Knappskog, David Wedge, James H.R. Farmery, Santiago Gonzalez, Inigo Martincorena, Ludmil B. Alexandrov, Peter Van Loo, Hans Kristian Haugland, Peer Kaare Lilleng, Gunes Gundem, Moritz Gerstung, Elli Pappaemmanuil, Patrycja Gazinska, Shriram G. Bhosle, David Jones, Keiran Raine, Laura Mudie, Calli Latimer, Elinor Sawyer, Christine Desmedt, Christos Sotiriou, Michael R. Stratton, Anieta M. Sieuwerts, Andy G. Lynch, John W. Martens, Andrea L. Richardson, Andrew Tutt, Per Eystein Lønning, Peter J. Campbell

**Affiliations:** 1 Wellcome Trust Sanger Institute, Hinxton CB10 1SA, UK; 2 Department of Clinical Oncology, Guys and St Thomas' NHS Trust, London SE1 9RT, UK; 3 Section of Oncology, Department of Clinical Science, University of Bergen, Bergen, Norway; 4 Department of Oncology, Haukeland University Hospital, Bergen, Norway; 5 Big Data Institute, University of Oxford, Oxford OX3 7BN, UK; 6 Cancer Research UK Cambridge Institute, University of Cambridge, Li Ka Shing Centre, Robinson Way, Cambridge CB2 0RE, UK; 7 European Bioinformatics Institute EMBL-EBI, Wellcome Genome Campus, Hinxton CB10 1SD, UK; 8 Theoretical Biology and Biophysics (T-6), Los Alamos National Laboratory, Los Alamos, NM 87545, USA; 9 Center for Nonlinear Studies, Los Alamos National Laboratory, Los Alamos, NM 87545, USA; 10 University of New Mexico Comprehensive Cancer Center, Albuquerque, NM 87102, USA; 11 The Francis Crick Institute, 1 Midland Road, London NW1 1AT, UK; 12 Department of Human Genetics, University of Leuven, 3000 Leuven, Belgium; 13 Department of Pathology, Haukeland University Hospital, Bergen, Norway; 14 The Gade Laboratory for Pathology, Department of Clinical Medicine, University of Bergen, Bergen, Norway; 15 Computational Oncology, Epidemiology and Biostatistics Memorial Sloan Kettering Cancer Institute, New York, NY 10065 USA; 16 Division of Cancer Studies, Faculty of Life Sciences and Medicine, King's College London, London SE1 9RT, UK; 17 Breast Cancer Translational Research Laboratory, Université Libre de Bruxelles, Institut Jules Bordet, Bd de Waterloo 121, 1000 Brussels, Belgium; 18 Erasmus MC Cancer Institute and Cancer Genomics Netherlands, Erasmus University Medical Center, Department of Medical Oncology, Rotterdam, the Netherlands; 19 Department of Pathology, Brigham and Women's Hospital, Boston, MA 02115, USA; 20 Dana-Farber Cancer Institute, Boston, MA 02215, USA; 21 Breast Cancer Now Research Unit, King's College London, London SE1 9RT, UK; 22 The Breast Cancer Now Toby Robins Research Centre, The Institute of Cancer Research, London SW3 6JB, UK

**Keywords:** breast cancer, metastasis, relapse, genomics, somatic mutation

## Abstract

Patterns of genomic evolution between primary and metastatic breast cancer have not been studied in large numbers, despite patients with metastatic breast cancer having dismal survival. We sequenced whole genomes or a panel of 365 genes on 299 samples from 170 patients with locally relapsed or metastatic breast cancer. Several lines of analysis indicate that clones seeding metastasis or relapse disseminate late from primary tumors, but continue to acquire mutations, mostly accessing the same mutational processes active in the primary tumor. Most distant metastases acquired driver mutations not seen in the primary tumor, drawing from a wider repertoire of cancer genes than early drivers. These include a number of clinically actionable alterations and mutations inactivating SWI-SNF and JAK2-STAT3 pathways.

## Highlights

• Metastases mostly disseminate late from primary breast tumors, keeping most drivers
• Drivers at relapse sample from a wider range of cancer genes than in primary tumors
• Mutations in SWI-SNF complex and inactivated JAK-STAT signaling enriched at relapse
• Mutational processes similar in primary and relapse; radiotherapy can damage genome

## Graphical Abstract

**Figure:**
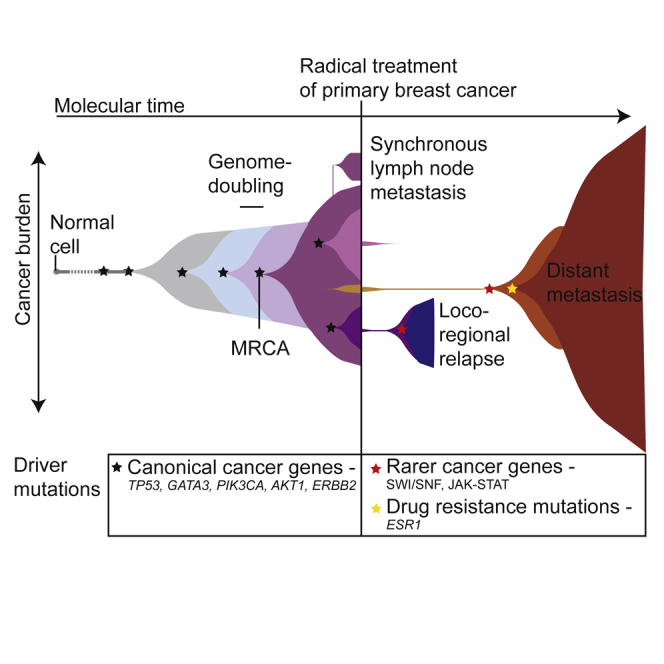

## Teaser

By sequencing primary, locally relapsed, and metastatic breast cancers, Yates et al. show that clones seeding metastasis or relapse disseminate late from primary tumors but continue to acquire mutations, including clinically actionable alterations and mutations inactivating the SWI/SNF and JAK2-STAT3 pathways.

## Significance

**These findings have implications for personalized therapy of breast cancer. The late dissemination of cells that seed metastasis or local relapse suggests that the primary tumor genome can proxy for the genome of disseminated cells at the time of first diagnosis, supporting the use of genome sequencing to aid decisions about adjuvant therapy for primary breast cancer. Biopsy and sequencing of metastases may be helpful in some patients because most distant metastases have acquired additional driver mutations not seen in the primary; these often involve potentially actionable genes and cellular pathways. Sequencing local recurrences can distinguish a genuine relapse from a second primary cancer, two scenarios with very different care pathways.**

## Introduction

Metastatic breast cancer is almost universally fatal within 5–10 years, a dismal statistic that has not changed much in the past 20–30 years (Tevaarwerk et al., 2013). Breast cancer recurrence can take two forms: distant metastasis (commonly bone, brain, liver, lung, and distant lymph nodes) and locoregional relapse (recurrence in breast, chest wall, or regional lymph nodes). Locoregional relapse occurs in about 10% of patients despite optimal management of the primary tumor and is associated with concomitant or future distant metastatic disease in 30% and 60% of cases, respectively. In contrast, regional lymph node metastasis found at the time of primary diagnosis is often cured with surgery and radiotherapy but is a well-established poor prognostic factor, associated with a higher risk of subsequent cancer recurrence.

Molecular profiling of breast cancer has typically focused on the primary breast lesion. Gene expression profiles classify breast cancers into different subtypes, with clinical trials showing that these transcriptional signatures can be used to support therapeutic decisions in primary breast cancer (Harris et al., 2016). Large-scale genomics analyses have now been performed in thousands of primary breast cancers, revealing the complex mutational landscape of the disease (Banerji et al., 2012, Cancer Genome Atlas Network, 2012, Ciriello et al., 2015, Ellis et al., 2012, Nik-Zainal et al., 2016, Shah et al., 2012, Stephens et al., 2012). General patterns to emerge from these studies include that estrogen receptor (ER)-positive primary breast cancer has a characteristic “luminal” transcriptional profile with frequent somatic mutations activating PI3K-AKT signaling and inactivating *GATA3* and the JUN kinase pathway. Breast cancers with amplification and/or overexpression of *ERBB2* (also known as *HER2*) have a distinct transcriptional and genomic profile, confirming the central role that *ERBB2* plays in the pathogenesis of this subtype of breast cancer. Breast cancers negative for ER, the progesterone receptor (PR), and HER2, so-called triple-negative breast cancers, are characterized by a “basal-like” transcriptional profile, frequent *TP53* mutation, and extensive copy number variation. A number of studies have revealed extensive genomic heterogeneity within primary breast tumors and changes in subclonal structure during systemic therapy (Balko et al., 2014, Gellert et al., 2016, Miller et al., 2016, Ng et al., 2015, Shah et al., 2012, Wang et al., 2014, Yates et al., 2015).

While the genome of primary breast cancer has been well characterized, there has been considerably less analysis of relapsed or metastatic breast cancer. Those studies that have been performed have revealed that metastases are clonally related to the primary tumor, sharing many of the driver mutations, but nonetheless have typically acquired additional variants not detectable in the primary lesion (Brastianos et al., 2015, De Mattos-Arruda et al., 2014, Ding et al., 2010, Hoadley et al., 2016, Juric et al., 2015, Savas et al., 2016, Shah et al., 2009, Yates et al., 2015). Due to small sample sizes, however, it has proved difficult to extract general patterns of evolution between primary and recurrence, leaving a number of unanswered questions with important biological and clinical implications. We conducted this study to address some of these questions, including how closely related a metastasis is to its primary lesion; whether there are differences in evolution across locoregional relapse, axillary metastases seeded by lymphatic spread, and distant metastases seeded by hematogenous spread; whether the driver landscape of metastases differs from primary cancers; and whether there are cancer genes specific to metastases. Since the survival of patients with metastatic breast cancer is so poor, it is particularly important to establish whether newly emerging driver mutations in the metastasis might offer opportunities for personalized therapy.

## Results

### Patient Cohort

**Figure 1:**
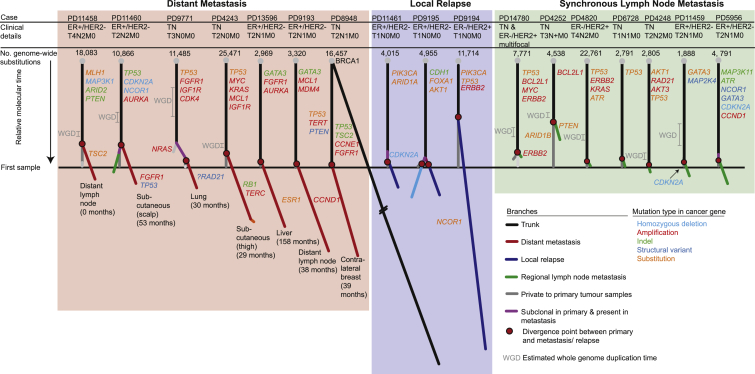
Phylogenetic Trees Describe Evolution of 17 Primary Breast Cancers to Metastasis or Local Relapse Each tree represents an individual patient's breast cancer inferred from the analysis of a matched normal sample and 2–4 tumor samples per case (total of 40 tumor samples). Trees are derived from genome-wide substitutions. Trees are grouped according to scenario: distant metastasis (red panel), locoregional relapse (blue panel), or synchronous axillary lymph node metastasis (green panel). Branches private to the metastasis or relapse follow the same color theme, while branches representing clones that are specific to the primary tumor are gray. The black trunk represents clonal mutations that are present in 100% of cells in every sample. Purple branches represent mutations within the metastasis or relapse that are subclonal within the primary tumor. Branch lengths reflect the proportion of clustered somatic mutations attributed to that subclone. The whole tree is scaled to the maximum length of a tree that would be inferred from mutations identified in the primary tumor. Red circles identify the point of divergence between the metastasis/relapse-seeding clone and the primary tumor. The estimated whole-genome doubling (WGD) time is indicated by 95% confidence intervals. Numbers in brackets reflect the months elapsed between primary tumor and metastasis sample acquisition. See also Figures S1 and S2 and Tables S1, S2, S3, S4, and S5.

The study comprises two major aims. In the first, to define patterns of genomic evolution between the primary cancer and disease progression, we performed whole-genome sequencing of 40 tumor samples from 17 patients to an average coverage of 42×, together with matched germline DNA samples (Tables S1 and S2). These 17 patients encompassed three clinical scenarios: synchronous axillary lymph node metastasis; distant metastasis and local relapse subsequent to definitive treatment for the primary tumor. In all but one case (PD11458), primary tumor samples were treatment naive and sampled at diagnosis. Metachronous recurrence samples were obtained 8–158 months after the primary tumor diagnosis. Distant metastatic samples were obtained from tumor deposits in lung (n = 1), liver (n = 1), distant skin regions (n = 2), contralateral breast (n = 1), and distant lymph nodes (n = 2) (Figure 1). All patients underwent standard management, including curative surgery with local radiotherapy, adjuvant anthracycline-containing chemotherapy, and/or endocrine therapies where appropriate (Table S1).

The second aim was to study the distribution of driver mutations in distant metastatic or locoregionally relapsed breast cancer. To achieve this, we analyzed 227 recurrence samples from 163 patients for point mutations and copy number changes in 365 known cancer genes to an average coverage of 467× (Tables S1 and S2). For 46 patients, 2–5 recurrence samples were sequenced, allowing heterogeneity of the recurrence landscape to be explored. For 51 individuals, the matched primary tumor was available for sequencing.

Samples were obtained in clinically relevant scenarios including first relapse or metastasis and following systemic treatment interventions. All samples therefore represent clinically progressing disease. Progression during a documented systemic therapy exposure occurred for 126 samples, including endocrine therapy (n = 43), anthracyclines (n = 45), taxanes (n = 12), and other chemotherapeutic regimens. Samples were obtained following a median of 2 systemic treatment exposures (range, 0–5) and after 39 months (range, 0–196) from primary cancer diagnosis (Table S1). Tumors were classified according to the primary tumor TNM stage, histological type, grade, and presence of estrogen receptor (ER), progesterone receptor (PR), and *ERBB2* (HER2) amplification.

### Evolution between Primary Breast Cancer and Metastasis/Relapse

Using whole-genome sequencing, we explored the patterns of genomic evolution in three clinical scenarios across 17 patients: local lymph node involvement at the time of primary tumor diagnosis (8 patients); locoregional relapse after apparently definitive primary tumor treatment (n = 4); and subsequent development of distant metastasis (n = 7) (Figure 1). We identified an average of 9,594 substitutions (range, 1,792–25,471), 1,098 indels (range, 60–12,786), and 245 structural variants (range, 6–786) within each individual's cancer genome (Table S3). We performed validation on 1,480 somatic substitutions and indels by custom capture pull-down or capillary sequencing (Table S4), confirming 1,436 (97%) were truly present and somatically acquired. We enriched our validation experiment with mutations that were private to one of the samples to enhance our ability to identify subclonal populations. Rearrangements were validated by the visual confirmation of breakpoint-associated copy number changes.

**Figure 2:**
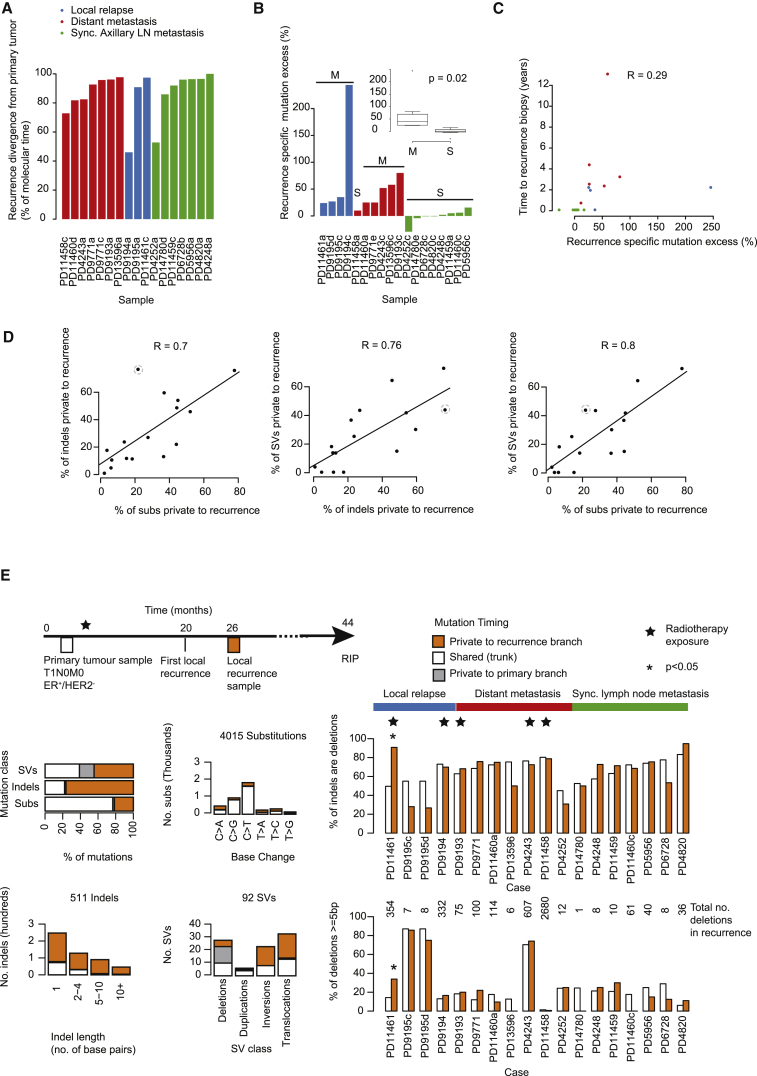
Genome-wide Somatic Mutation Timing in 16 Breast Cancers (A) For each of 17 primary tumor samples, the bar height reflects the point in molecular time that the recurrence seeding clone is estimated to diverge from the primary tumor (relates to phylogenetic trees in Figure 1). Molecular time is determined from the number of base substitutions. (B) The recurrence-specific mutation excess is reported in a barplot for each of 18 recurrence samples and in a boxplot split by synchronous (S) and metachronous (M) cases, where the box represents the interquartile range (IQR) bisected by the median, whiskers represent the maximum and minimum range of the data that do not exceed 1.5× the IQR while outlier data points extend beyond this. The recurrence-specific mutation excess indicates the base substitution load in branches private to the recurrence minus those in branches private to the primary tumor, presented as a percentage of all substitutions identified in the primary tumor. The p value is generated by an F test. (C) The recurrence-specific mutation excess as presented in (B) according to the time from primary tumor diagnosis and acquisition of the relapse sample, each dot represents a patient. R = Pearson's correlation coefficient. (D) Scatterplots compare the proportion of each the major mutation types, indels (insertions and deletion), substitutions (Subs), and structural variants (SVs), localized to the recurrence. Unlike (A) and (B), these figures include variants in regions that were variable in copy number across samples. (E) Radiation mutation signature at relapse following external beam radiation. The mutation spectrum of an outlier sample (PD11461) highlighted by a dashed gray circle in (D) is shown in detail. The overall contribution of indels and structural variants (SVs) outweighs that of substitutions at relapse (top left barplot). Within this sample, indels of greater lengths (bottom left barplot) and inversions and translocations (bottom, middle bar plot) are relatively more common after relapse. Cohort-wide, the relative contribution of deletions as opposed to insertions (top right barplot) and of deletions of 5 base pairs (bp) or longer (bottom right barplot) are reported.^∗^p < 0.0001 (Fisher's exact test) for enrichment in the relapse sample. Cases exposed to prior external beam radiotherapy are indicated by a star symbol indicating that other samples do not seem to carry the same signature. See also Figure S3.

To reconstruct the phylogenetic structure underlying disease progression, we applied bioinformatic and deductive reasoning approaches, as described previously (Nik-Zainal et al., 2012, Yates et al., 2015) (Figures 1, S1, and S2A; Table S5). We used multi-dimensional Bayesian Dirichlet processes to cluster somatic substitutions from multiple related samples according to their respective mutation burden, corrected for tumor cellularity, allele-specific copy number, and regions of differential chromosomal deletion between samples. We identified an average of 2.8 distinct clusters per patient (48 in 17 patients), with 94% of these reproduced by independent clustering of high-coverage targeted validation data (Figure S1 and Table S5). Individual clusters inform on the structure of the phylogenetic tree, typically enabling a single “tree solution” to be derived for each case. In 16 of the 17 cases, all samples studied were clonally related, as demonstrated by thousands of shared somatic mutations, with the trunk of the phylogenetic tree representing 12%–98% of all clustered somatic substitutions (Figures 1 and 2A, Table S5).

One critical observation emerges: the genomic landscape of the primary breast cancer at diagnosis is a good surrogate for the somatic mutations present in disseminated cells at that moment in time. This conclusion derives from several aspects of the data. First, the metastatic or relapsing clones branch late from the phylogenetic lineage of the primary breast lesion, with relatively few mutations private to the primary tumor. On average, metastatic divergence occurs at 87% of molecular time within the primary tumor, estimated from phylogenetic analysis of base substitutions in regions of the genome with the same copy number across all lesions (Figure 2A and Table S5). Second, as expounded in more detail in the next section, the excess mutational burden of metachronous metastasis or relapse clones exceeded that of synchronous axillary lymph node metastases (p = 0.02, F test) and the one synchronous distant metastasis (PD11458) (Figure 2B). Indeed, synchronous lymph node metastases are typically very similar to the primary breast lesion (green branches, Figure 1). Third, driver mutations tend to be concentrated on the trunk of the phylogenetic tree, notwithstanding the 1 or 2 additional driver events acquired by relapse or metastasis clones (explored in considerable detail in later sections) (Figure 1). Furthermore, whole-genome duplication, when present, precedes the branching of the recurrence clone (Figures 1 and S2B). Finally, as we shall see, the mutational processes active on the trunk of the phylogenetic tree tend to persist in the metastasis, suggesting that inferences (and therapeutic decisions) based on mutational signatures in the primary will extend to the unseen disseminated cells.

One patient (PD8948), a germline *BRCA1* mutation carrier, was diagnosed with a triple-negative cancer of the left breast, and over the next 10 years, treated for two apparent local relapses of this lesion and a distant metastasis to the contralateral breast. In fact, our genomic analyses revealed that the three lesions affecting the left breast were clonally unrelated, completely independent primary cancers, with the second of them seeding the contralateral breast metastasis (Figure S3). This is important clinically as the management and prognosis of a second primary cancer and a local relapse are distinct. This case demonstrates that genome sequencing can clarify the nature of presumed local “relapses,” especially important in individuals with a genetic predisposition to breast cancer.

Taken together, then, these patterns of disease evolution strongly support the use of genome sequencing of the primary breast cancer lesion to underpin decisions about systemic therapy in the adjuvant setting. In modern breast cancer treatment, the major aim of chemotherapy or estrogen suppression is to kill those cells that have already spread from the primary lesion, since surgery and local radiotherapy are usually sufficient to cure the primary. If it were the case that relapsing or metastatic clones disseminated early from the primary breast cancer with extensive parallel evolution, as has been suggested previously (Klein, 2009), then targeting somatic mutations found in the primary would not necessarily have much relevance to disseminated cells without those changes.

### Additional Burden of Mutations in Relapse Samples

For patients with synchronous axillary lymph node metastases, the number of mutations private to the metastasis was broadly equivalent to the number private to the primary cancer (Figures 1 and 2B). This is perhaps not surprising since, by virtue of being synchronous lesions, the major lineages in the primary and the metastasis had the same time period in which to accrue mutations after divergence. In contrast, for the local relapse and metachronous distant metastasis samples, the relapse carried, on average, 63% more mutations than the primary tumor, albeit with considerable variability among patients (range, 24%–244% extra). The number of additional mutations in the relapse only loosely correlated with the time elapsed between diagnosis of the primary cancer and relapse (Pearson's correlation R = 0.29) (Figure 2C).

The additional mutation burden in the later relapse sample was substantially greater than the chronological time elapsed between primary and metastasis would suggest, implying that the rate at which mutations accumulate has typically increased during breast cancer evolution. Strikingly, we find that the fraction of additional substitutions, indels, and structural variants in the relapse sample compared with the primary tumor are broadly in concert with one another (Pearson's correlation R = 0.7–0.8; Figure 2D). One consequence of the continued structural variation is that deletions of genomic regions add to the diversity of point mutations between subclones.

One patient (dotted circle, Figure 2D), however, had distinctly more indels in the relapse sample than would be suggested for the number of additional base substitutions. This sample was from a local relapse, occurring 2 years after a small, node-negative primary cancer treated with wide local excision and adjuvant radiotherapy. More than 90% of the indels at relapse were deletions rather than insertions, compared with <50% of indels on the trunk of the phylogenetic tree for that patient (odds ratio [OR] = 11.5; p = 1 × 10^−21^; Fisher's test) or 67% in all other patients' cancers (OR = 3.4; p = 1 × 10^−14^; Fisher's test; Figure 2E). The deletions occurring at relapse were typically longer than those in the primary tumor, with 33% being 5–100 bp in size versus 14% in the primary (OR = 3.1, p = 0.03; Fisher's test; Figure 2E). We recently described the signature of small to medium-sized deletions as a characteristic feature of radiation-induced secondary cancers (Behjati et al., 2016). This suggests that in this patient, the relapsing clone was exposed to adjuvant radiotherapy and survived, albeit with genomic damage from the ionizing radiation. In contrast, in other relapse samples from patients treated with adjuvant radiotherapy, this signature was not evident (Figure 2E), perhaps suggesting that the cells that ultimately seeded these relapses had already disseminated outside the radiation field.

**Figure 3:**
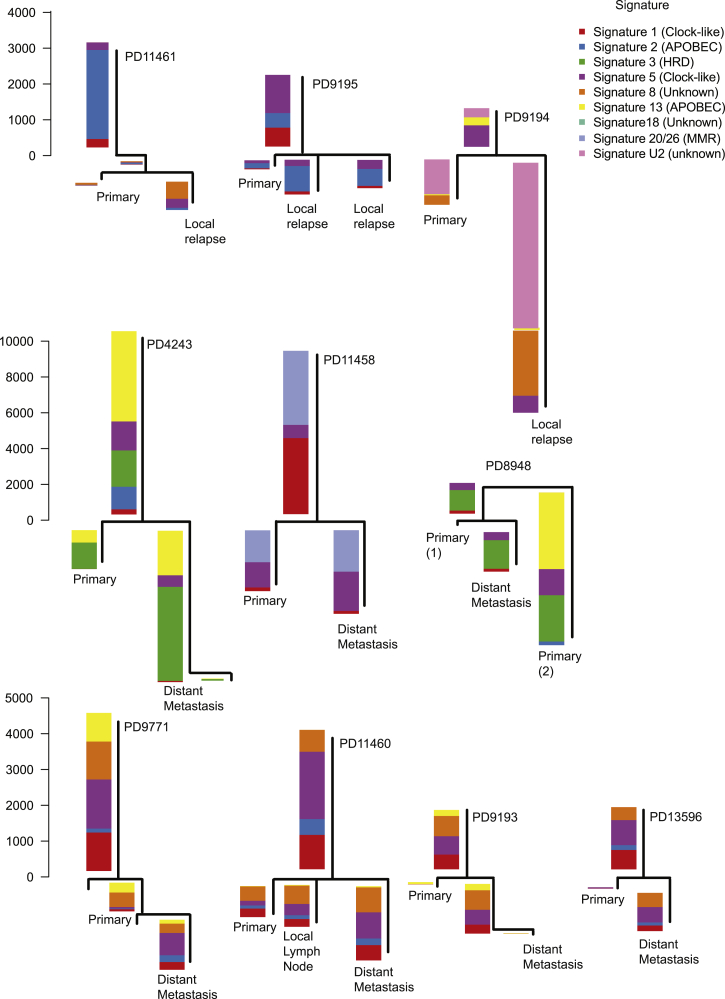
Genome-wide Mutation Signatures in Ten Metastatic or Locally Relapsed Breast Cancers Annotated to Phylogenetic Trees The mutational signature composition of each phylogenetic tree branch is reported for the ten multi-sample, whole-genome cases with a local relapse or distant metastatic sample. HRD, homologous recombination deficiency; MMR, mismatch-repair deficiency. See also Figure S4.

To assess which mutational signatures are most significant at different stages of disease evolution, we examined their relative contributions to each branch of the phylogenetic tree (Figure 3). Perhaps the most striking feature is that the heterogeneity in mutational signatures across patients is considerably greater than the heterogeneity across different evolutionary stages within a given tumor. This suggests that a given breast tumor accesses only a subset of the mutational processes potentially available to it, but those mutational processes contribute genomic variation on an ongoing basis. Nonetheless, there are some shifts in the relative contributions of mutational processes over time. The universal signature of C > T transitions at CpG dinucleotides (signature 1) contributes a relatively higher proportion of mutations early in disease evolution, likely because this signature is relatively constant throughout life and gets swamped by processes emerging later in disease evolution. Mutations attributed to the activity of APOBEC enzymes, characterized by C > T and C > G variants in a TpC context (signatures 2 and 13), were rather variable in their timing, being predominantly early in some patients (such as PD11461), more prominent in late stages in others (PD9195), and relatively steady in many (PD4243) (Figure 3). These patients had a range of systemic cytotoxic treatments following their primary cancer diagnosis, including anthracyclines, cyclophosphamide, and 5-fluorouracil; the lack of new signatures in relapsing lesions suggests that these chemotherapeutic agents are not major drivers of mutation accumulation.

### Telomere Integrity during Cancer Evolution

We estimated telomere lengths from whole-genome data for germline and tumor samples. Telomere lengths showed variation among individuals and across samples from the same individual (Figure S4 and Table S5). Greater variability of telomere lengths was seen among tumors (mean = 7,703 bp; range, 2,409–27,621 bp) compared with germline samples (mean = 6,627 bp, range, 4,351–11,077 bp) (Figure S4B). There was no simple relationship between telomere length and the number of somatic substitutions, indels, or structural variation within tumor samples (Figure S4C).

In six of the eight cases where we sequenced breast tumors and adjacent normal breast epithelium, the telomere was shorter in the primary tumor, suggestive of telomere attrition during cancer development. Between primary tumor and recurrence samples within a patient, there was no consistent pattern, with telomeres sometimes lengthening, sometimes shortening. A few samples had especially long telomeres; one was associated with amplification of *TERT* (telomerase reverse transcriptase) and another with amplification of *TERC* (telomerase RNA template component).

### Driver Mutations Are Acquired during Cancer Progression

For each tumor, we manually curated the driver mutations among the set of breast cancer genes known to be recurrently targeted by point mutations (Kandoth et al., 2013, Lawrence et al., 2014), structural variants, and copy number changes (Beroukhim et al., 2010). We found that most driver mutations occurred in the primary tumor and were located on the trunk of the phylogenetic tree (Figure 1). Among the nine cancers that underwent whole-genome duplication, all driver mutations arose prior to this event, indicating that they are usually relatively early events in cancer evolution.

Among the synchronous lymph node metastases, only one patient had a driver mutation (in *PTEN*) seen in the metastasis that was not present in the primary tumor, confirming that there is generally little genomic divergence between primary and synchronous local lymphatic metastases. In one case (PD11460), we analyzed both a distant metastasis and a synchronous local lymph node metastasis, finding that the lymph node deposit was more closely related to the primary tumor than the subsequent distant metastasis and did not contain any private driver mutations (Figure 1). This observation is consistent with the highly divergent pattern recently reported between regional lymph node metastases and brain metastases (Brastianos et al., 2015).

**Figure 4:**
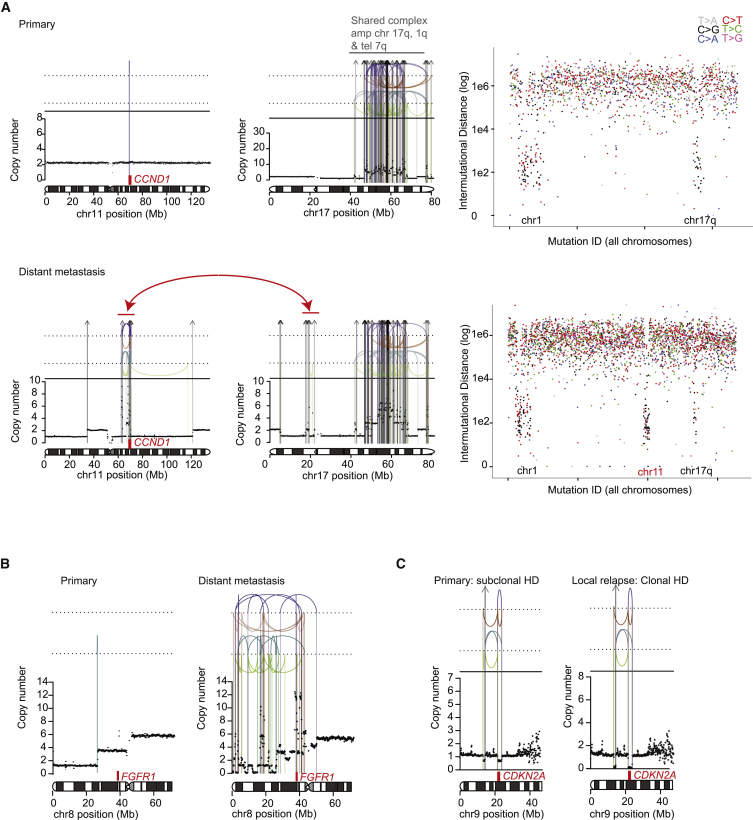
Structural Variant Driver Mutations at Relapse in Three Breast Cancers (A) Case PD9193: De novo amplification of *CCND1* in a distant lymph node metastasis. Structural variant breakpoints are represented by colored vertical lines: interchromosomal translocations (gray arrows), tail-to-tail inversions (green), head-to-head inversions (blue), tandem duplications (orange), deletions (purple). Rainfall plots report the inter-mutational distance of individual consecutive mutations where each dot reflects a mutation and the color represents the base change. (B) Case PD11460: de novo amplification of *FGFR1* in a metastatic deposit. (C) Case PD11461: a subclone containing a homozygous deletion in *CDKN2A* in the primary tumor seeds a local relapse.

Five of seven WGS-analysed patients with distant metastases, however, had one or two additional driver mutations specific to the metastasis sample, suggesting that growth of the metastatic clone in its new niche is abetted by further genomic evolution. We observed several instances of complex clusters of structural variants that were acquired late in the major metastasis lineage. These included an event that generated a complex amplification of *CCND1* coupled with loss of one copy of *TP53* (Figure 4A) and a chromothripsis (Stephens et al., 2011) event that resulted in *FGFR1* amplification (Figure 4B). Interestingly, these data showing complex, catastrophic events during metastasis development echo recent single-cell sequencing studies showing punctuated copy number evolution in primary breast cancer lesions (Gao et al., 2016).

Of the three locoregional relapse cases, one relapse that branched from the primary tumor particularly early in molecular time acquired a new driver mutation in *NCOR1*. Another arose from a subclone in the primary tumor that carried a homozygous deletion of *CDKN2A*; this event became fully clonal in the relapse (Figure 4C).

Thus, these data show that distant metastasis and locoregional relapses are typically associated with acquisition of additional driver mutations compared with the primary tumor, whereas driver mutations in synchronous lymph node metastases are typically also present in the primary.

### The Driver Landscape of Relapse and Metastasis

To provide more complete statements about the landscape of driver mutations at breast cancer recurrence, we performed sequencing of all coding exons of 365 known cancer genes in 227 samples from distant metastases or locoregional relapses across 163 patients. The primary tumor was available for 51 of these patients and germline DNA for 81. For comparison, we also interrogated these genes from sequenced exomes of 705 primary breast cancers published by the TCGA, which we reanalyzed using the same pipeline as for our cohort (Table S3).

Samples that were from local relapses or metastases harbored a higher number of driver point mutations on average than those in the primary tumor cohort (2.0 versus 1.6; p = 0.0008; F test). In 25 (49%) of the 51 patients from whom we analyzed the matched primary tumor, a driver mutation was found that was private to the relapse sample. This was more pronounced for distant metastases; a driver mutation not found in the primary lesion was seen in 74% of distant metastases compared with 29% of locoregional relapses (p = 0.002, Fisher's test).

**Figure 5:**
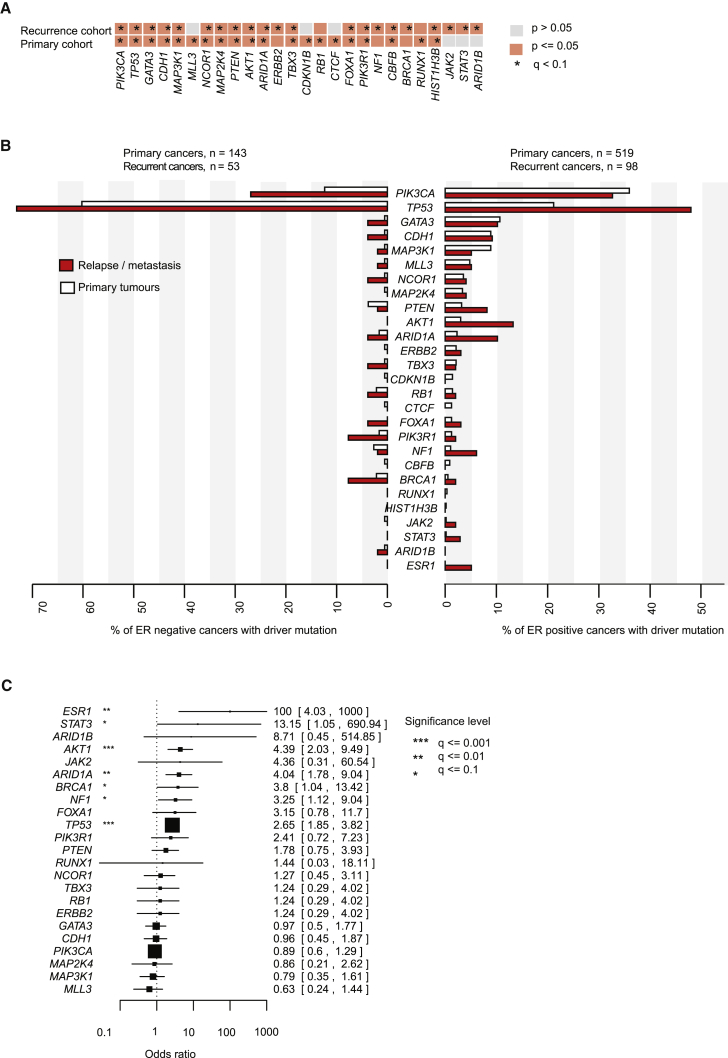
Comparison of the Driver Landscapes of 163 Recurrent and 705 Primary Breast Cancers (A) Cancer genes identified as significantly mutated with a false discovery rate (q) < 0.1, applied to the TCGA 705 primary breast cancer exomes or the 163 recurrent breast cancers independently. (B) Barplots compare the prevalence of each significantly mutated cancer gene and *ESR1* in the primary and recurrent breast cancer cohorts (662 and 151 cases, respectively, where the estrogen receptor status of the primary tumor was documented). (C) Forest plot comparing the frequency with which cancer genes are mutated in the relapse cancer cohort (163 cases) compared with the primary tumor cohort (705 cases). Enrichment for each gene was determined using two-sided Fisher's exact tests and Benjamini and Hochberg correction. Box size is scaled to the number of cases and whiskers, and numbers inside brackets represent the 95% confidence interval for the odds ratio (the upper limit is clipped at 1,000).

We compared the rate of non-synonymous mutations with synonymous mutations across the 365 genes. This technique, well established for inferring selection in comparative genetics, was adapted for somatic mutations (Martincorena et al., 2015), taking account of the trinucleotide composition of the genes, gene size, mutation spectrum, and local variation in mutation rates across the genome. A total of 21 and 20 cancer genes were identified as significantly mutated (false discovery rate, q < 0.1) in the primary and relapse cohorts, respectively, of which 15 genes were significant in both cohorts (Figure 5A). We note that *BRCA1* and *NF1* were not significant in the primary cancer cohort after correction for multiple hypothesis testing, something we believe to be due to the play of chance given the wealth of data implicating these two genes in primary breast cancer. When split by whether tumors were ER-positive or ER-negative, we found that most breast cancer genes showed higher rates of driver mutation in the relapse/metastasis samples than in primary tumors (Figure 5B). The exception to this was *PIK3CA* and *MAP3K1* in ER-positive tumors, in keeping with reported better relapse-free survival rates in primary breast cancers carrying *PIK3CA* mutations.

We formally tested whether each gene was significantly more frequently mutated in relapsed or metastatic breast cancer than primary breast cancer (Figure 5C). In general, ORs were skewed toward greater enrichment in relapse or metastatic samples, reflecting the greater number of driver mutations and the wider repertoire of genes mutated. Significant differences for individual genes were not detected among locoregional relapses compared with distant metastases.

### Driver Mutations Acquired Late Encompass a Wider Range of Cancer Genes

**Figure 6:**
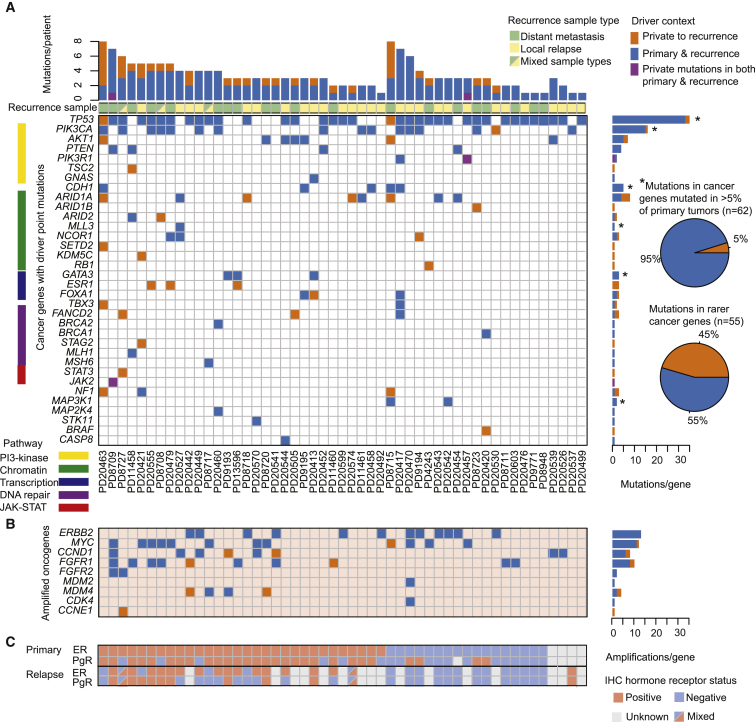
Temporal Distribution of Mutated Cancer Genes in 51 Paired Primary Tumor and Relapse Samples (A) The heatmap indicates if the driver mutation is early (blue), defined as present in both the primary tumor and recurrence, or late, being detected in the recurrence deposit(s) only (orange), or different mutations in the same gene seen in both the primary and recurrence (purple). Asterisks (^∗^) indicate cancer genes mutated in >5% of 705 primary tumor samples. The pie charts compare the proportion of mutations that are private to recurrence samples within most commonly mutated genes and within comparatively rare cancer genes (mutated in <5% of primary tumors). Stacked barplot above the heatmap relates cumulative incidence of point mutations and amplifications in (C) for each individual patient. (B) Temporal ordering of amplified oncogenes derived from analysis of next-generation sequencing data. Tile colors follow the format stated in (A). (C) Blue and pink tiles indicate the immunohistochemical (IHC) classification by estrogen receptor (ER) and progesterone receptor (PgR) of primary and relapse samples, where a split tile indicates multiple relapse samples with different ER/PgR statuses. See also Figures S5 and S6.

There are two possible explanations for the enrichment of driver mutations in relapse/metastasis samples compared with the cohort of primary breast cancers. It might be that those primary breast cancers with a more disordered genome are more likely to subsequently relapse; or it might be that the relapsing clone continues to acquire new driver mutations after dissemination from the primary lesion. We therefore compared the driver mutation profile of the 51 patients in whom both the primary and a relapse/metastasis sample were sequenced (Figures 6A–6C).

Mutations in well-known, relatively frequent breast cancer genes, such as *TP53*, *PIK3CA*, and *GATA3*, when present, were typically found in both the primary and the recurrence samples. In contrast, mutations in less frequent cancer genes were often found only in the recurrence. This pattern was particularly striking for genes involved in SWI/SNF signaling, such as *ARID1A*, *ARID1B*, and *ARID2*, which were commonly wild-type in the primary lesion but inactivated in the recurrence (Figures 6A, S5, and S6). This echoes recent data from metastatic endometrial cancer (Gibson et al., 2016), locally progressive hepatocellular carcinoma (He et al., 2015), and a pan-cancer metastasis study (Zehir et al., 2017), where mutations in these same genes are also acquired late in disease evolution.

In primary breast cancer, ER-positive and triple-negative tumors show rather distinct combinations of driver mutations, with *PIK3CA*, *GATA3*, and MAPK-pathway mutations characterizing the former and *TP53* and copy number alterations the latter. When studying relapse and metastasis samples, however, we found that the genomic differences between triple-negative and ER-positive cancers became more blurred: *TP53* mutations were seen in 40%–50% of relapsed ER-positive cases; and *PIK3CA*, *GATA3*, *CDH1*, and *MAP3K1* all increased several-fold in relapsed ER-negative cancers. We identified ER and PgR expression loss in 17% and 41% of cases, respectively, across the relapsed breast cancer cohort (Figure 6C). Loss of ER expression at relapse was frequently associated with driver mutations in *TP53* (90% of cases) and *ARID1A* (30% of cases). While *TP53* mutations were usually early events, detected in the primary tumor, *ARID1A* was more often private to the relapse sample in association with hormone receptor loss (Figures 6A, 6C, and S5).

### Late JAK-STAT Pathway Inactivation

**Figure 7:**
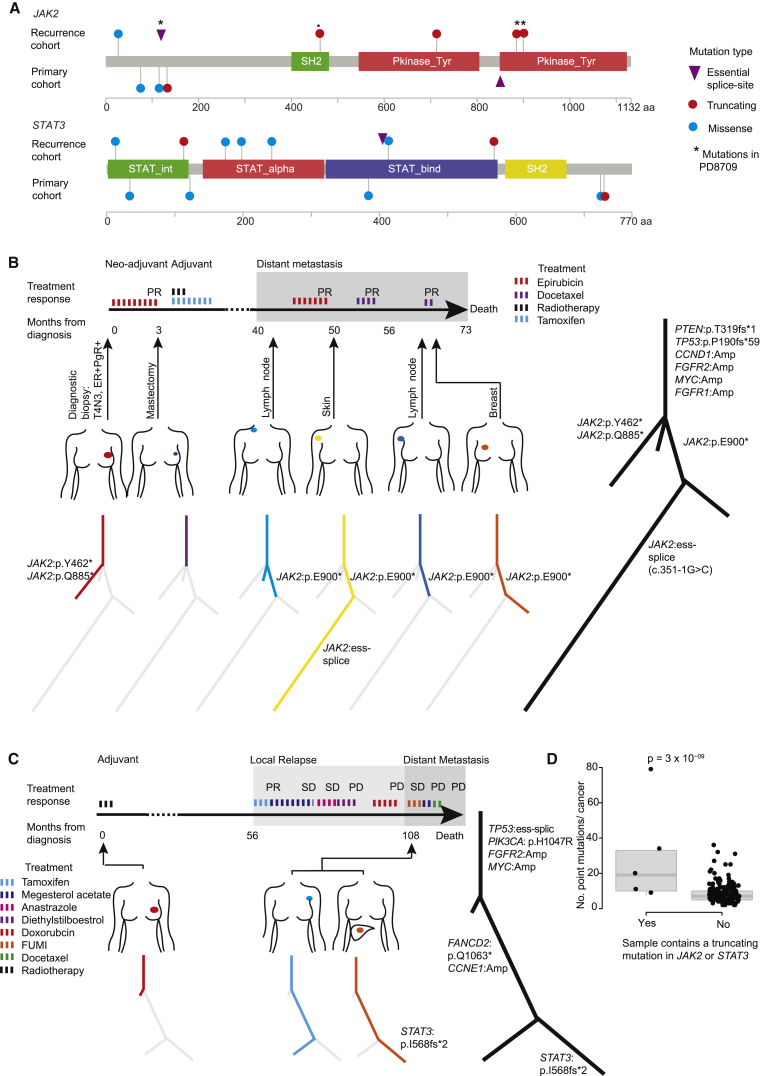
JAK-STAT Inactivating Mutations Are Enriched at Relapse (A) Pencil plots of *JAK2* and *STAT3* genes annotated with non-synonymous mutations identified in the relapse cohort (n = 163) and the primary cohort (n = 705). (B) A case (PD8709) of parallel evolution involving four truncating mutations in *JAK2*. Response to treatment exposures are documented. SD, stable disease; PR, partial response; PD, progressive disease. (C) A case (PD8727) of *STAT3* truncating mutation arising in a liver metastasis. (D) Scatter and boxplot of the number of mutations identified in samples (within the relapse cohort, 163 cases) that do or do not contain a *JAK2* or *STAT3* truncating mutation. The box represents the interquartile range (IQR) bisected by the median, whiskers represent the maximum and minimum range of the data that do not exceed 1.5× the IQR. p value generated using an F test.

Interestingly, *JAK2* and *STAT3* were identified as significantly mutated in the metastasis/relapse screen even though they had not been discovered in the earlier (and larger) exome studies of primary breast cancers (Banerji et al., 2012, Cancer Genome Atlas Network, 2012, Ellis et al., 2012, Shah et al., 2012, Stephens et al., 2012). Both showed an excess of protein-truncating mutations, such as nonsense base substitutions, frameshift indels, and essential splice site mutations (Figure 7A), suggesting that they are operating as tumor suppressor genes in breast cancer. All such mutations in this cohort arose in ER-positive cancers in contrast to *JAK2* amplifications that have been identified in triple-negative cancers (Balko et al., 2016). One patient showed an especially remarkable example of parallel evolution of inactivating *JAK2* mutations (Figure 7B). During this tumor's evolution, four different *JAK2* inactivating mutations occurred, all on subclonal branches of the phylogenetic tree, with several of the lesions apparently having compound heterozygous inactivation of the gene. This is reminiscent of the frequency of parallel evolution of resistance to PARP inhibitors in ovarian cancers through *BRCA1/2* reversion mutations (Patch et al., 2015).

*JAK2* and *STAT3* mutations both showed a trend toward being more frequent in distant metastasis samples (Figure 5C), which may explain why these were detected as significant in our study but not in previous studies of primary breast cancer. For example, in one patient who had a local relapse followed by a liver metastasis, the liver metastasis carried a *STAT3* inactivating mutation that was absent from both the primary cancer and the local relapse, despite the latter being closely related to the metastasis (Figure 7C).

Thus, inactivation of JAK-STAT signaling appears to contribute to disease progression and metastasis in some patients with breast cancer. We note that in another study of metastatic breast cancer, a *JAK2* nonsense mutation was also discovered (Zehir et al., 2017). Interestingly, homozygous loss of *JAK2* has recently been described as a mechanism of resistance to checkpoint inhibitor immunotherapies (Zaretsky et al., 2016), probably acting through blocking the interferon-gamma pathway. Although none of the patients here received such therapies, it is feasible that these mutations help advanced tumors evade the native immune response mounted against them. Cancers with *JAK2* or *STAT3* truncating mutations contained a higher number of point mutations on average than other cancers (p = 3 × 10^−9^; F test, Figure 7D). Although other explanations are possible, this finding would be consistent with the notion that these cancers may contain more neoantigens, stimulating a more exuberant native immune response, and driving selection of JAK-STAT pathway inactivation.

### Treatment Exposures Influence Breast Cancer Evolution

The broadening of the repertoire of cancer genes sampled by late driver mutations likely reflects the diverse selective forces operating during evolution of advanced breast cancer. These include selective pressures exerted by therapeutic interventions, by the immune system responding to the expansion of a clone carrying many neoantigens, and by the very different microenvironment in a metastatic site compared with breast epithelium.

A total of 139 samples of recurrent disease were taken shortly before a systemic treatment was commenced (Figure S6A), of which 59 displayed progressive disease, indicating treatment resistance, and 80 cancers stabilized or responded to treatment (Table S1). Across all treatments, *TP53* and *ESR1* driver mutations were more frequent in cases that progressed (63% in progression cases versus 45% in stable disease, p = 0.04; and 7% versus 0%, p = 0.03 respectively; Fisher's exact test), as seen in a recently published series of metastatic breast cancers (Zehir et al., 2017). Mutant *TP53* has previously been associated with endocrine and anthracycline resistance (Aas et al., 1996, Berns et al., 1998). Gain-of-function mutations in *TP53* have been associated with metastasis and drug resistance in cell-line and xenograft models (Petitjean et al., 2007, Turner et al., 2017), but in our cohort loss-of-function and gain-of-function mutations were equally enriched in patients with progressive disease compared with stable disease (p = 0.5; Fisher's exact test) (Figure S6B), and in recurrences compared with primary tumors (p = 0.7; Fisher's exact test) (Figure S6C). As previously reported, *ESR1* resistance mutations were found in five patients previously treated with endocrine therapies (Chandarlapaty et al., 2016, Robinson et al., 2013, Toy et al., 2013) and predicted progressive disease upon switching treatment (Figure S5).

Truncating mutations in SWI-SNF cancer genes, including *ARID1A* and *ARID2*, emerged in three of five cancers relapsing after taxane chemotherapy (Figure S6D). Interestingly, an association between loss of *ARID1A* expression and chemoresistance has also been observed in clear-cell ovarian cancers (Katagiri et al., 2012).

A handful of potentially actionable driver mutations emerged during endocrine therapy (Figures S5 and S6D). These included amplifications of the *MDM4, FGFR1*, and *CCND1* oncogenes in two patients each, with an additional patient acquiring a canonical *BRAF* V600E mutation. *FGFR1* activation and *TP53* pathway inactivation (including *MDM2/4* activation) have previously been associated with endocrine resistance (Ellis et al., 2012, Turner et al., 2010). The implication here is that oncogene amplification or activation may represent a common mode of breast cancer evolution in the face of endocrine therapy. Since oncogenes are more natural therapeutic targets than tumor suppressor genes, this raises the interesting possibility of new personalized interventions for some patients relapsing after endocrine therapy.

## Discussion

The concept of precision oncology is founded on the presumption that knowing the genomic basis of a patient's cancer will guide choice of targeted therapies likely to be efficacious. This rests on the key assumption that we can obtain a sample representative of the tumor cells that we are targeting with that therapy. In patients diagnosed with primary breast cancer, systemic therapy is aimed at killing the microscopic deposits of cells that have disseminated from the breast, as surgery and radiotherapy will generally cure the primary lesion. In the samples studied here, we found that at the time of initial diagnosis, the genome of the primary would have been a good proxy for that of the cells that ultimately seeded the relapse, whether the spread be local or via a hematogenous or lymphatic route. In particular, the vast majority of driver mutations found in the primary cancer would also be present in the relapsing clone. Our observation of late dissemination is consistent with the findings of a recent study that combined bulk sequencing of primary tumors with single-cell sequencing of bone marrow-derived disseminated tumor cells, the presumed precursor of clinically overt metastatic disease (Demeulemeester et al., 2016). Although the genome of a metastatic clone may be similar to the primary tumor at first diagnosis, by the time it has expanded to be clinically detectable, extensive further genomic changes have occurred.

Whether patients presenting with distant metastatic disease should have that metastasis biopsied or not to decide on therapeutic interventions is a controversial question (Arnedos et al., 2015). Many sites of metastatic disease are challenging and invasive to sample, demonstrated by the bias seen in our cohort toward sites of disease that are easy to access. Our data indicate that metastases seeded by hematogenous spread do continue to evolve after dissemination, acquiring many new somatic mutations and key driver mutations. A recent study from a large tertiary referral unit has shown that many patients with metastatic breast cancer are willing to undergo biopsy of recurrent lesions for molecular profiling (Zehir et al., 2017).

In its restless search for a genome ideally suited to autonomous life in far-flung regions of the body, a breast cancer can access many different mutational processes and a wide repertoire of cancer genes. The result is considerable patient-to-patient variability in genomic profiles, even more pronounced than the already daunting levels seen in primary breast cancer. Mapping this complexity will require recruitment of large, prospective cohorts of patients with metastatic disease and integration with transcriptional, epigenomic, and clinical readouts. Our data show that such an endeavor would have potential clinical impact, providing insights into patterns of clonal evolution, mechanisms of therapy failure, and pathways that could represent new therapeutic targets.

## STAR★Methods

### Key Resources Table

**Table:** 

REAGENT or RESOURCE	SOURCE	IDENTIFIER
**Deposited Data**		

Targeted and whole genome sequence data	https://www.ebi.ac.uk/ega/	Accession numbers: Targeted (2939stdy) EGAD00001002698 Exome (492stdy): EGAD00001002697 Whole genome (2040stdy): EGAD00001002696
Somatic Mutation Calls	Mendeley Data	http://dx.doi.org/10.17632/g7kpzkhz8c.1

**Software and Algorithms**		

The Sanger’s Cancer Genome Project core somatic calling workflow from the ICGC PanCancer Analysis of Whole Genomes (PCAWG) project	https://dockstore.org/containers/quay.io/pancancer/pcawg-sanger-cgp-workflow	

### Contact for Reagent and Resource Sharing

Further information and requests for resources and reagents should be directed to and will be fulfilled by the Lead Contact, Peter J Campbell (pc8@sanger.ac.uk).

### Experimental Model and Subject Details

#### Subjects, Samples and Consent

All samples included in this project were obtained with informed patient consent and handled in line with the wider framework and approval for the Breast Cancer Genome Analyses for the International Cancer Genome Consortium Working Group led by the Wellcome Trust Sanger Institute, Cambridgeshire, UK, REC reference: 09/H0306/36. We performed MPS and analysis of a total of 299 tumor samples collected from 170 individual’s breast cancers and 87 matched normal, germline samples (Table S1). Three patients were male and the remainder female, the average age at primary tumor diagnosis was 53 years (range 30-85 years). Clinical details including tumor stage, histological features and hormone receptor status are summarized in Table S1. Clinical follow-up data was available for 96% of patients. The cohort reflects a very poor prognostic group of patients whereby 96% of these patients were diagnosed during their disease course witheither distant metastatic disease (86%), very poorly controlled locoregional disease not amenable to surgical resection (10% of cases) or both (7%).

#### Whole Genome ‘Triplet’ Cohort

To permit phylogenetic analysis of the progression from primary tumor to metastasis a total of 39 fresh frozen tumor samples were collected from 17 females and subjected to whole genome sequencing. Samples were obtained from Dana-Farber Cancer Institute, Boston, US (7 cases); Kings College Hospital, London, UK (4 cases); The Erasmus MC Cancer Center, Rotterdam, The Netherlands (4 cases); The Institute Jules Bordet, Brussels, Belgium (2 cases) in line with local ethics committee approvals (project SHARE” #93-085, approved by the Dana-Farber Harvard Cancer Center institutional review board; UK, REC reference: 10/H0804/33, approved by Guy’s and St Thomas’ NHS Trust ethics committee; MEC 02.953, approved by the medical ethical committee of the Academisch Ziekenhuis Rotterdam (EUR/ AZR) for ‘The retrospective assessment of cell biological factors in archival tumor tissues’; Protocol 1698 and 1634, approved by the Institut Jules Bordet local ethics committee). For each individual, in addition to a primary tumor and a matched normal sample at least one sample from a distinct metastatic scenario was included in the experiment. For 7 individuals, where the metastasis scenario sample was limited to a synchronous lymph node deposit these samples were only included in the whole genome analysis where they form a comparison cohort. For one patient (PD8948) where the apparent relapse samples were identified as distinct primary tumors we collected two additional tumor samples from formalin fixed paraffin embedded (FFPE) tissue blocks and performed targeted capture on both samples and whole genome sequencing on one (Figure S3).

#### Relapsed Breast Cancer Cohort

The number of cases of locally relapsed and distant metastatic breast cancer was extended from 10 to 163 by including a second cohort of patients for whom 365 cancer related genes were sequenced using a targeted capture pulldown approach. Samples from these individuals form the ‘relapsed breast cancer cohort’. The additional 153 patients were drawn from a single centre study at the Department of Oncology, Haukeland University Hospital, performed with the aim of identifying genetic alterations in advanced and metastatic breast cancer deposits. Between March 1996 and October 2004, a total of 206 patients with non-operable primary breast cancers, local relapse and / or metastatic deposits suitable for biopsy were recruited to the study. All samples were snap-frozen in liquid nitrogen in the operating room immediately upon removal from the patient. We analysed a total of 259 tumor samples from 153 patients. Patients included in the analysis had at least one sample from a distant metastatic or locoregional relapse deposit that contained sufficient material for DNA extraction, allowing MPS. For 41 patients included in the study we were able to identify a primary tumor sample and extract sufficient DNA for MPS (FFPE, n = 29; fresh frozen, n = 12). In addition, DNA was retrieved from FFPE-blocks from four metastatic deposits undergoing routine biopsy in the time period between primary and fresh frozen metastatic tissue collection, 2 primary tumors after neo-adjuvant chemotherapy and a synchronous lymph node deposit. The study was approved by the regional ethics committee of the Norwegian Health Region West (218/97 – 77.97; REK Vest), and all patients provided written informed consent.

In total, 163 individuals were therefore included within the relapsed breast cancer cohort and for each patient at least one sample (total number of relapse samples = 227) was obtained from a distant metastatic deposit (n = 79) or a metachronous loco-regional relapse (n = 148) (Table S1). Multiple relapse samples were collected for 46 individuals (range 2-5 samples per individual). A matched primary tumor sample was collected in 51 cases and a matched germline sample was collected for 80 cases (adjacent normal breast tissue, n = 6; blood, n = 74). The distribution of relapse sample sites is presented in Table S1. Most (177/227) relapse and metastasis samples were pre-treated, being exposed to an average of 1.7 (range 0-5) lines of systemic therapy Table S1. A total of 80 samples from 57 individuals were also obtained after exposure to external beam radiotherapy.

#### Primary Breast Cancer (Comparison) Cohort

The primary tumor comparison cohort consisted of previously published exome data from 705 individual’s primary breast cancers, freely available from The Cancer Genome Atlas (TCGA). We included properly matched samples that were available for download from CGHub on December 2015. We excluded variants where the matched normal coverage was lower than 10-fold and samples for which less than 50% of the mutations detected by our calling pipeline were present in the somatic mutation calls released by TCGA. To minimize bias in our comparisons we applied the same mutation calling algorithms, post-processing filters and driver annotation processes as were used for in in-house generated data for the relapse cohort. Annotated mutation data for these samples, within the scope of the cancer gene panel is available in Table S3. Clinical information for the 705 patients in the primary cohort was downloaded from https://tcga-data.nci.nih.gov/docs/publications/brca_2012/ (file = BRCA_Clinical.tar.gz ). A comparison of the clinical characteristics of the primary cohort and relapse cohort at the point of diagnosis is provided in Table S1. Cancer staging information for each dataset was determined using the American Joint Committee on Cancer (AJCC) Staging Manual, 7^th^ edition. When nodal status was recorded as ‘Nx’ within clinical information this is assumed to reflect node negative disease (‘N0’).

### Method Details

#### Sample Size

The sample size of 163 recurrent breast cancers has 99% power that a cancer gene mutated in 5% of breast cancer recurrences would be seen in at least 3 patients in the cohort.

#### Tumor Specimen Processing

All samples within the whole genome and relapse breast cancer cohorts were histopathologically assessed to ensure adequate tumor cellularity (>=70%) and if necessary macrodissection was performed. Where possible for both primary tumor and relapse samples ER and PgR expression was determined by local pathologists as Allred scores of 4 or above. Where available, HER2 over-expression was determined by IHC scores of 3+ or 2+ confirmed by fluorescent in-situ hybridisation. Due to the historical nature of the Haukeland University Hospital sample set, HER2 expression data however, is scarce and HER2 amplification was determined from sequence data using the criteria for identifying amplifications in targeted capture data as described below. We have previously shown our approach to yield results that are highly consistent with clinical HER2 status results (Yates et al., 2015).

#### DNA Extraction

DNA from fresh frozen tumor tissue specimens and blood samples was isolated, using spin columns from the QIAamp DNA mini kit (Qiagen). The procedure was performed according to the manufacturer’s instructions with the exception that 400ul sample (instead of 200ul) was used as input in the cases where full blood on EDTA were used instead of leukocyte concentrates (Haukeland University Hospital cases). DNA from formalin fixed paraffin embedded tissue (FFPE) was isolated, using spin columns from the QIAamp DNA FFPE Tissue Kit (Qiagen). The procedure was performed according to the manufacturer’s instructions, with the following exceptions: The de-paraffinization step with xylene was repeated three times and the subsequent washing step with ethanol was repeated twice. Lysis of tissue was performed using 540 μl buffer ATL and 60 μl proteinase K per samples, for 2-4 hours at 56°C, before addition of a further 180 μl buffer ATL and 20 μl proteinase K and an over-night incubation at 56°C.

#### Multi-Sample Whole Genome Sequencing

Genomic libraries with insert sizes of 300bp-600bp were derived from native DNA from 39 tumor and 17 matched normal fresh frozen samples using Illumina® paired end sample preparation kits according to manufacturers instructions. Following cluster generation, 100bp paired-end sequence data was generated using Illumina HiSeqs and was subsequently aligned to the reference human genome (NCBI build37) using BWA. Whole genome libraries from a single FFPE tumor (PD8948c) and matched fresh frozen normal sample (PD8948b) were prepared using Agilent Technologies Sure Select library preparation kit (Custom library kit (cat no. 930075) http://www.agilent.com/search/?Ntt=930075 following manufacturers instructions. 150bp paired end sequence data (with average insert sizes of 319bp and 481bp respectively) was generated using Illumina X10. The average genome wide sequence coverage of tumors and matched normal samples was 42 and 31 fold respectively (Table S2).

#### Multi-Region Targeted Gene Screen

For targeted capture pulldown experiments we used a bait design that consisted of over 8,000 targets of which almost 6,000 covered the exons of 365 genes. To facilitate copy number analyses baits were also included to target over 2,000 SNPs outside of exonic locations. Genomic DNA from tumor and matched normal samples, was fragmented using Covaris® (average insert size ∼150bp) and subjected to Illumina® DNA sequencing library preparation using Agilent’s® Bravo Automated liquid handling platform. Tumor and normal samples were indexed with unique barcodes using PCR. Libraries were then hybridised to custom ribonucleic (RNA) baits according to the Agilent® SureSelect® protocol. Samples were multiplexed on average 16 samples per lane and flow-cell clusters created. Paired-end, 75bp sequence reads were generated using Illumina HiSeq 2000®. Sequence data was re-aligned to the human genome (NCBI build 37) using BWA. Unmapped reads, PCR duplicates and those outside of the target region were excluded from analysis. The average sequence coverage of tumors and matched normal samples was 467 and 505 fold respectively (Table S2).

#### Multi-Sample Mutation Calling

Substitutions, indels and structural variant breakpoints were called independently in each tumor sample using mutation calling algorithms (CaVEMan, Pindel and BRASS) and post-processing filters as previously described (Yates et al., 2015). Mutation calling algorithms used in the analysis are freely available at https://github.com/cancerit/. Where an individual had more than one tumor sample we performed a comparative analysis of SNP and indel variant data for union of sites from all related samples in an unbiased manner using in-house software – vafCorrect, that is freely available at https://github.com/cancerit/vafCorrect. For substitutions unbiased pileup results were obtained using Bio::DB::HTS (https://github.com/Ensembl/Bio-DB-HTS). For indels the approach includes unmapped reads whose pair is mapped within the vicinity (defined by library insert size) of the indel site and resulting reads were aligned using exonerate to original reference sequence and alternate reference sequence (created by inserting the indel variant at the given reference location). Exonerate output was then parsed to count the fraction of reads aligned to original reference and alternate reference sequence. Reads that were mapped with equal identity scores to reference and alternate sequence were reported as ambiguous reads while reads that were present at the variant location but did not map to either of the reference sequences were categorized as unknown reads. Data quality was ensured and the impact of germline SNP contamination minimized by filtering against an extended unmatched normal panel of over 200 samples, cross-referencing with available germline SNP databases, using a matched normal sample where available and visually inspecting local alignments for all reported coding mutations.

Comprehensive lists of all somatic substitutions, indels and structural variants from whole genome analysis are available for download at review@sftpsrv.sanger.ac.uk. All high confidence mutation calls within the scope of the cancer gene panel are presented in Table S3.

#### Mutation Validation

For the whole genome experiment, native DNA where available (25 tumor and 14 normal samples) or whole genome amplified (WGA) DNA when necessary (samples PD13596a, PD13596b, PD13596c, PD4243a, PD4252c, PD48102a, PD8948d, PD8948e) was subjected to custom capture pulldown and high depth re-sequencing to a target depth of 1000-fold. Probes were designed for 6,534 genome-wide substitutions and indels using Agilent Technologies SureSelect Standard DNA Design Wizard. High-stringency repeat masking, a tiling density of 2X and balanced boosting were applied to the design. DNA capture (paired-end, average insert size 150bp) libraries were multiplexed and sequenced using Illumina MiSeq® to an average coverage of 1,076-fold. We have previously published validation data for case PD9771 (Yates et al., 2015). To determine an experimental validation rate, all coding indels (n=144) and substitutions (n=1,498) were included in the experiment. A true positive validation of 94% was identified for both coding indels and substitutions independently. Amongst substitutions the most common reason for failure to validate was low coverage (4%) and this was usually associated with the use of WGA material. Excluding WGA validation experiments was associated with a validation rate of 97% and this is believed to be a more reliable reflection of the true positive rate (Table S4).

The remaining variants included in the high-depth pulldown design were selected to enable validation and refinement of phylogenetic tree structures. The approach was biased towards subclonal events and mutations that contradicted the consensus tree. Mutations close to each other or within 200bp of germline snps were also enriched to permit reconstruction of the subclonal structure through phasing approaches. Using this approach, across 13 cases (cases PD13596, PD4252, PD4820, PD114780 excluded for reasons stated above), 45 out of 48 tree branches were identified through an independent clustering experiment, re-capitulating and therefore validating the basic tree structure in each case. A total of 3 small branches failed to validate and consisted of 2-3% of the overall mutation burden in each case. See Figure S1 and Table S5 for details of phylogenetic tree construction and validation in each case.

Regarding multi-sample targeted capture experiments we have previously demonstrated a 99% consistency rate in reporting mutation presence and absence (Yates et al., 2015) using custom pull down duplicate experiments. Furthermore, we validated non-synonymous mutations in *ESR1, JAK2* and *PIK3R1* using capillary sequencing (Table S4). One *ESR1* mutation was validated in an independent exome experiment (Brown, 2017).

#### Cancer Gene Discovery

To identify recurrently mutated driver genes we used dNdScv, as previously described in detail (Nik-Zainal et al., 2016). This method uses dN/dS and covariates to detect genes with higher density of non-synonymous coding mutations than expected by chance. The method considers the trinucleotide mutation spectrum, the sequence of each gene, the impact of coding mutations (synonymous, missense, nonsense, splice sites substitutions and indels) and the variation of the mutation rate across genes. Multiple testing correction (Benjamini-Hochberg FDR) was applied across analyzed genes and a q value < 0.1 was used to determine statistical significance. For the relapse cohort significance was tested across the 311 genes for which at least one mutation was called. The approach was performed across all relapse samples and across the subset of samples with a matched germline sample. *STAT3* was significantly mutated in the matched sample analysis only. Within the exome analysis over 20,000 genes are analyzed.

#### Driver Mutation Annotation

Each coding variant was manually curated with a likely driver status following a systematic approach. Firstly, likely cancer genes were identified as either those found within the dNdS cancer gene discovery approach described above, from published reference materials consisting of the Cancer Gene Census (Futreal et al., 2004), the Cancer5000 series (Lawrence et al., 2014) or from literature review of breast cancer sequencing studies (Banerji et al., 2012, Cancer Genome Atlas Network, 2012, Ellis et al., 2012, Shah et al., 2012, Stephens et al., 2012). Subsequently, oncogenic mutations were annotated within these cancer genes. Oncogenic mutations were defined as those falling into one of the following categories: 1) A canonical oncogenic mutation in a recurrent mutation hotspot; 2) A lower frequency recurrent mutation in a known oncogene with 3 or more confirmed somatic non-synonymous substitutions or in-frame deletions previously reported at this locus in COSMIC or confirmed through experimental models or special cohorts (i.e. *ESR1* resistance mutations); 3) Likely damaging events in a known tumour suppressor that include truncating (nonsense), frame-shift, essential splice variants or those within a mutation hotspot (>=2 somatic mutations); 4) Silent mutations in a known recurrent splice site hotspot.

#### Genome-Wide Subclonal Copy Number Analyses

Segmental copy number information was derived from all targeted capture and whole genome data using the Allele Specific Copy Number Analysis of Tumors (ASCAT) algorithm (Van Loo et al., 2010). The Battenberg algorithm was used to identify clonal and subclonal copy number changes in whole genome sequence data as previously described (Nik-Zainal et al., 2012, Yates et al., 2015) and was also used to challenge and confirm copy number and ploidy estimates derived from ASCAT. The approach phases germline SNPs within MPS data using Impute2 (Howie et al., 2009) that uses a well characterized panel of polymorphic SNPs.

Within whole genome data, copy number segments are reported as amplified when present at more than twice the estimated average ploidy across the whole genome. Homozygous deletions are identified as segments where total copy number equals zero or equivalent in an area of subclonal copy number. Within targeted capture data the mean logR and 95% confidence interval was calculated across known cancer driver genes. Potential amplifications in common breast cancer genes were identified based on a mean logR of > 1, equating to 6 alleles in a diploid genome and tumor cellularity of 50%. For related samples where heterogeneity of amplification events was called logR and BAFs across all genes and point mutation data were reviewed manually in each related sample to determine if heterogeneity is likely a consequence of low aberrant cell fraction as opposed to true driver heterogeneity. This conservative approach was adopted to minimize the risk of over-calling heterogeneity.

#### Genome-Wide Multi-Sample Clonality Analyses

For the 17 patients with multi-sample whole genome sequencing data, to model the subclonal structure across multiple related samples previously described bioinformatics and deductive reasoning approaches were adopted. The approach follows 3 main steps including the identification of large-scale subclonal copy number changes using the Battenberg algorithm (Nik-Zainal et al., 2012), clustering of subclonal somatic substitutions using a Bayesian Dirichlet process in multiple dimensions across related samples and hierarchical ordering across multiple samples using the ‘pigeon hole principle’. Strict quality control is applied to the mutations included in clustering analysis to avoid the generation of false positive clusters of mutations:

• During evolution, copy number losses may result in the loss of mutations in the affected regions, resulting in clusters of mutations found uniquely in the unaffected sample(s). In order to avoid falsely calling such mutations as arising from a clonal expansion in the unaffected samples, such mutations are excluded from Dirichlet process clustering.
• Some mutations may be present in multiple samples, but only called in a subset of samples, due to low allele frequency in the other sample(s). To avoid false negatives, allele frequencies of all mutations found in any sample from a patient are therefore re-called, with a minimum mapping quality and base quality of 10.
• The allele frequencies of all mutations are adjusted to cancer cell fraction using purity and copy number information. Copy number segments have start and end points defined by heterozygous SNP locations, so somatic variants that fall between these boundaries have undefined copy number and are excluded from clustering.

A median of 95% (range 77 – 99%) of mutations are included in clustering. Using this approach each substitution that passed quality control was assigned to a specific cluster (Tables S5). For each individual case, data including cluster size (equating to phylogenetic tree branch length), cluster ‘position’ (reflecting the proportion of cells containing the mutation cluster in each related sample) and posterior confidence intervals are presented for both discovery and validation experiments in Table S5 and Figure S1.

#### Mutation Timing in Multi-Sample Analyses

The relative contribution of the different mutation types during evolution (Figure 2) was estimated by comparing the proportion of mutations that were shared, private to the primary tumor sample or private to the metastasis/ relapse sample. Each individual point mutation was assigned to one of these categories by calculating for each mutation, in all related samples independently, R’s pbinomial statistic based upon a conservative, expected error rate of 1 in 200. A mutation was deemed to be present or absent from an individual sample based upon a p value of <=0.05 or > 0.05 respectively. All structural variants reconstructed in silico were determined to be shared or private to the primary/ metastasis samples based upon either reconstruction in related samples or the presence of 4 or more split reads supporting the breakpoint using BRASS1. Substitution branch timing (Figures 1 and 2) was calculated using mutation clustering where the cluster size dictates the branch length.

#### Whole Genome Duplication Timing Analysis

In this study we have estimated the prevalence of 3 different developmental stages for 22 of the breast cancer samples. The first one corresponds to the diploid stage previous to whole genome duplication. The second one is the tetraploid cell stage after the whole genome duplication was acquired and previous to the subclonal diversification. Lastly, the timing between the last selective sweep and the emergence of the detected subclones. The duration of each of the stages in molecular time is estimated via the fraction of mutations having arisen in each of the phases. To estimate the proportions of mutations in each stage we employ a strategy similar to that of Purdom et al. (Purdom et al., 2013) and extend it to subclonal mutations.

Let *ρ* denote the purity of the sample. The expected variant allele frequency f_i_ for a mutation arising in state I depends on the number mutated alleles m_i_, the total copy number c (4 in our case) and the prevalence of the subclone p_i_. For early clonal mutations we have p_i_ = 1 and m_i_ = 2, for late clonal mutations we have m_i_ = 1. For subclonal mutations we have p_i_ < 1 and m_i_ = 1.

$$f=\frac{\rho \;m_{i}p_{i}}{4\rho +2\left(1-\rho \right)}$$

We model the number of reads X arising from a mutation in stage I as a binomial with coverage n.

$$X|\;i\;∼\;Binom\;\left(n,\;f_{i}\right)$$

The probability that a mutation occurs in stage I is π_i_. This gives rise to a binomial mixture model.

$$P\left(X,\;i\right)=P\left(X\;\text{|}\;i\right)\times \pi_{i}$$

Using Bayes’ formula we can compute the probability of being in state I given X as

$$P\left(i\;\text{|}X\right)=\frac{P\left(X,i\right)}{P\left(X\right)}=\frac{P\left(X\;\text{|}\;i\right)\times \pi_{i}}{\sum_{i}P\left(X\;\text{|}\;i\right)\times \pi_{i}}$$

For a series of k observed mutations with variant reads x_1_, … x_k_, we can estimate the mixture proportions π_i_ using and EM algorithm.

Knowing the probabilities π_i_, for early (π_e_) and late (π_l_) stages we can calculate an estimate the relative time of WGD as:

$$t=\frac{2\;\pi_{e}}{2\;\pi_{e}+\pi_{l}}$$

To assess the robustness of the above estimator and to calculate confidence intervals we use bootstrapping, subsampling 100 times from the number of observed mutations with replacement and calculating t for each of the subsamples.

Presented analyses were first applied to all mutations within individual samples with results being consistent with duplication arising prior to primary-relapse divergence. A more accurate estimate of the timing of whole genome duplication was then determined by restricting the analysis to shared, clonal mutations allocated to the trunk of the phylogenetic tree.

#### Driver Mutation Enrichment Analyses

The frequency with which each cancer gene (*ESR1* or genes significantly mutated in the driver discovery experiment) was altered by a driver mutation was compared between the relapsed and primary breast cancer cohorts using a two-sided Fishers’s exact test. A Benjamini-Hochberg correction for multiple testing was applied to generate false discovery rates (q). A total of 7 genes were significantly enriched in the relapsed compared to the primary cohort (defined by q < 0.1) while no genes were enriched in the primary cohort.

#### Mutational Signature Analysis

We assessed the relative activity of mutational processes over time by allocating somatic mutations to their specific branch of the phylogenetic tree and subjecting individual branches (composed of more than 20 mutations) to mutational signature analysis (Figure 3). Mutational signatures were detected in two independent ways: (i) *de novo* extraction based on somatic substitutions and their immediate sequence context and (ii) refitting of previously identified consensus signatures of mutational processes. The *de novo* extraction was performed using a previously developed theoretical model and its corresponding computational framework (Alexandrov et al., 2013b). Briefly, the algorithm deciphers the minimal set of mutational signatures that optimally explains the proportion of each mutation type in each mutational catalogue and then estimates the contribution of each signature to each sample. Within this dataset the computational framework identified five reproducible mutational signatures that closely resembled previously identified breast cancer signatures.

In the second stage, 27 distinct consensus mutational signatures previously identified from examining 7,042 samples across 30 different cancer types were ‘refitted’ (Alexandrov et al., 2013a). All possible combinations of up to seven mutational signatures were evaluated for each sample. This resulted in 1,285,623 solutions per sample and a model selection was applied to select the optimal solution. The model selection framework excludes any solution in which a mutational signature contributes less that 2% of the somatic mutations or less than 50 somatic mutations. Exceptions were made for Signatures 1 and 5 as these are believed to reflect on-going endogenous mutational processes that continuously contribute very low numbers of somatic mutations (Alexandrov et al., 2013a). Further, the model selection framework selects the solution that optimizes the Pearson correlation between the original pattern of somatic mutations and the one based on refitting the sample with consensus mutational signatures such that each additional signature should improve the Pearson correlation with at least 0.02. The final solution for each sample contained between 3-6 mutational signatures and these signatures were consistent with the ones previously identified by the *de novo* analysis: Signature 1, Signature 2, Signature 3, Signature 5, Signature 8, and Signature 13.

#### Telomere Length Estimates

Telomerecat is a *de novo* method for the estimation of telomere length from whole genome sequencing samples. The algorithm works by comparing the ratio of complete telomere reads to reads on the boundary between telomere and subtelomere. The ratio is transformed to a measure of length using a simulation approach that takes into account the fragment length distribution of the sample. By considering the ratio of complete telomere reads to boundary reads, Telomerecat estimates coverage over the telomere without interface from the affects of aneuploidy, a common occurrence in cancer. Telomerecat also corrects for error in sequencing reads by modeling the observed distribution of phred scores associated with mismatches to the telomere sequence.

#### Case PD8948 and Whole Genome Sequencing and Analysis of an FFPE Sample

For all but one cancer in the dataset we found that thousands or tens of thousands of somatic substitutions were shared by the primary and metastasis sample. In one case (PD8948) however, we determined from the clonal mapping of over 16,000 somatic substitutions, indels and structural variants that the two fresh frozen DNA samples from tumors in the left and right breast (samples PD8948d and PD8948e respectively) sampled 1 year apart were clonally unrelated cancers. Only 95 (0.6%) point mutations were detected in both samples, none of which fell within coding regions, and validation through visual inspection and/ or targeted capture pulldown failed to identify any mutation as a true positive in both samples. Copy number profiles and structural variant profiles from the two cancers were also distinct. In the absence of shared somatic events we conclude that these samples are derived from 2 independent primary tumors. The samples however, shared thousands of germline SNPs and a *BRCA1* frame-shift mutation confirming that they are derived from the same individual who was a known germline *BRCA1* mutation carrier.

To further explore the clonal evolution of this patient’s cancers we identified 2 additional FFPE samples from earlier tumor deposits within the left breast (PD8948a and PD8948c). These samples were subjected to targeted gene panel sequencing and the likely phylogenetic relationships between the four samples were then inferred from coding non-synonymous mutations as demonstrated in Figure S3A. The findings were consistent with the patient having developed 3 separate primary tumors during her lifetime, each containing a distinct *TP53* mutation. Two samples (PD8948c and PD8948e) harbored identical *TP53* (p.Y220C) and *KDM6A* mutations suggesting that the later sample represented distant relapse. Genome-wide analysis could provide conclusive evidence to confirm the relatedness of two such samples, however one sample (PD8948c) was derived from a 7 year old FFPE sample and there is little experience of whole genome sequencing of FFPE derived tumors. Weand others have previously shown low error rates for gene capture and whole exome sequencing of FFPE samples but to date we are only aware of the results from a single tumor sample sequenced to whole genome level and the widespread applicability of this single case is unclear. We predicted that the process of fixation and storage of such material could result in the introduction of technical artifacts and could compromise mutation-calling sensitivity. However, for the purposes of this experiment identifying a significant overlap of mutations called in the later sample (PD8948e) would confirm relatedness of the samples.

Library preparation of the FFPE sample was performed following our standard protocol and the tumor sample and a matched blood derived normal sample were sequenced to 31X and 38X respectively using Illumina X10®. Mutations were called using the same algorithms as previously described. Confirming the clonal relationship of the two samples, a significant proportion of somatic mutations of all classes – substitutions (25%), indels (18%) and structural variants (14%), were shared. This equates to almost 2,000 common somatic mutations, genome-wide (Figure S3B).

Analysis of other whole genome triplet cases within the cohort identified that all metachronous samples contain a significant excess point mutation burden compared to the primary tumor. However, this was inconsistent in this case, where the presumed primary tumor (PD8948c, FFPE) and the relapse sample obtained 3 years later (PD8948e, fresh frozen) contained a similar private point mutation burden (Figure S3B). We investigated whether the unexpected excess of FFPE specific mutations was a likely biological or technical phenomenon by comparing the mutation spectra in the 2 samples. The substitution profile within the FFPE sample indicated an excess of C>A base changes and these tended to occur in the context of one or two 5 prime cytosine nucleotides (Figure S3C). To investigate this further we applied formal mutational signature analysis to the private and shared branches from an inferred phylogenetic tree derived from these samples (Figure S3D). The analysis confirmed that the shared mutations were drawn from 3 mutation signatures – two clock-like signatures and a dominant signature associated with homologous recombination deficiency (signature 3), consistent with the known BRCA1 mutation carrier status. Private mutations identified in the fresh frozen sample (PD8948e) followed an almost identical signature distribution. In contrast, none of the mutations that were private to the FFPE sample were assigned to these signatures, but rather were purely assigned to a mutation signature (R2) - a known sequencing artifact that arises due to oxidative damage and has previously been described in relation to exome library preparation (Costello et al., 2013). In constructing the phylogenetic tree in Figure 1 we therefore omit a private to primary tumor branch, although it is conceivable that a small number of true private mutations were undetected due to the presence of an overwhelming artifact.

### Quantification and Statistical Analysis

Statistical analysis was performed and graphics produced using R version 3.0.1: A language and environment for statistical computing (R Foundation for Statistical Computing, Vienna, Austria. Alignment viewing was performed using Gbrowse®, Jbrowse®, Samtools® tview and IGV®. All hypothesis tests were 2-sided when appropriate and statistical tests used are specified in Results and figure legends.

### Data and Software Availability

Targeted and whole genome sequence data has been deposited at the European Genome-Phenome Archive (https://www.ebi.ac.uk/ega/ at the EBI) with accession numbers:

• Targeted (2939stdy) EGAD00001002698;
• Exome (492stdy): EGAD00001002697;
• Whole genome (2040stdy): EGAD00001002696.

Full somatic mutation calls (substitutions, indels and structural variants) for each individual cancer analyzed by whole genome sequencing are available for download from **Mendeley Data**. The link for the dataset is: http://dx.doi.org/10.17632/g7kpzkhz8c.1

The most recent version of our whole genome sequencing mutation pipeline is available as a Docker image. This, together with documentation, can be accessed from https://dockstore.org/containers/quay.io/pancancer/pcawg-sanger-cgp-workflow.

## Author Contributions

Conceptualization, L.R.Y., P.J.C., P.E.L., S.K., and A.T.; Methodology, L.R.Y., P.J.C., P.E.L., S.K., D.W., I.M., L.B.A., P.V.L., M.G., S.G., M.R.S., E.S., E.P., A.G.L., and J.H.R.F.; Software, D.W., I.M., L.B.A., P.V.L., L.R.Y., G.G., M.G., S.G.B., K.R., D.J., A.G.L., and J.H.R.F.; Histopathological analysis, H.K.H., P.K.L.; Validation, L.R.Y., S.K., L.M., C.L.; Resources, A.M.S., J.W.M., A.L.R., P.E.L., C.S., C.D., P.G., L.R.Y., and A.T.; Data curation, L.R.Y., S.K., P.E.L.; Visualization, L.R.Y.; Writing – Original Draft, L.R.Y. and P.J.C. Writing – Review and Editing, all authors; Supervision, P.J.C. and P.E.L.

## Supplemental Information

Document S1. Figures S1–S6

Table S1. Clinical Data, Related to Figure 1

Table S2. Sequencing Metrics, Related to Figure 1

Table S3. Mutation Calls, Including Targeted Capture Data for Primary and Relapse Cohorts, Related to Figure 1

Table S4. Validation Experiments, Related to Figure 1

Table S5. Somatic Mutation Clusters, Related to Figure 1

Document S2. Article plus Supplemental Information
