# Supplementary material for: Genomic Evolution of Breast Cancer Metastasis and Relapse
Source: Cancer Cell. 2017 Aug 14;32(2):169–184.e7. doi: 10.1016/j.ccell.2017.07.005 (PMC5559645; doi:10.1016/j.ccell.2017.07.005)
Supplement: Document S1. Figures S1–S6 [file mmc1.pdf]

Cancer Cell, Volume *32*

Supplemental Information

Genomic Evolution of Breast Cancer Metastasis and Relapse

Lucy R. Yates, Stian Knappskog, David Wedge, James H.R. Farmery, Santiago Gonzalez, Inigo Martincorena, Ludmil B. Alexandrov, Peter Van Loo, Hans Kristian Haugland, Peer Kaare Lilleng, Gunes Gundem, Moritz Gerstung, Elli Pappaemmanuil, Patrycja Gazinska, Shriram G. Bhosle, David Jones, Keiran Raine, Laura Mudie, Calli Latimer, Elinor Sawyer, Christine Desmedt, Christos Sotiriou, Michael R. Stratton, Anieta M. Sieuwerts, Andy G. Lynch, John W. Martens, Andrea L. Richardson, Andrew Tutt, Per Eystein Lønning, and Peter J. Campbell

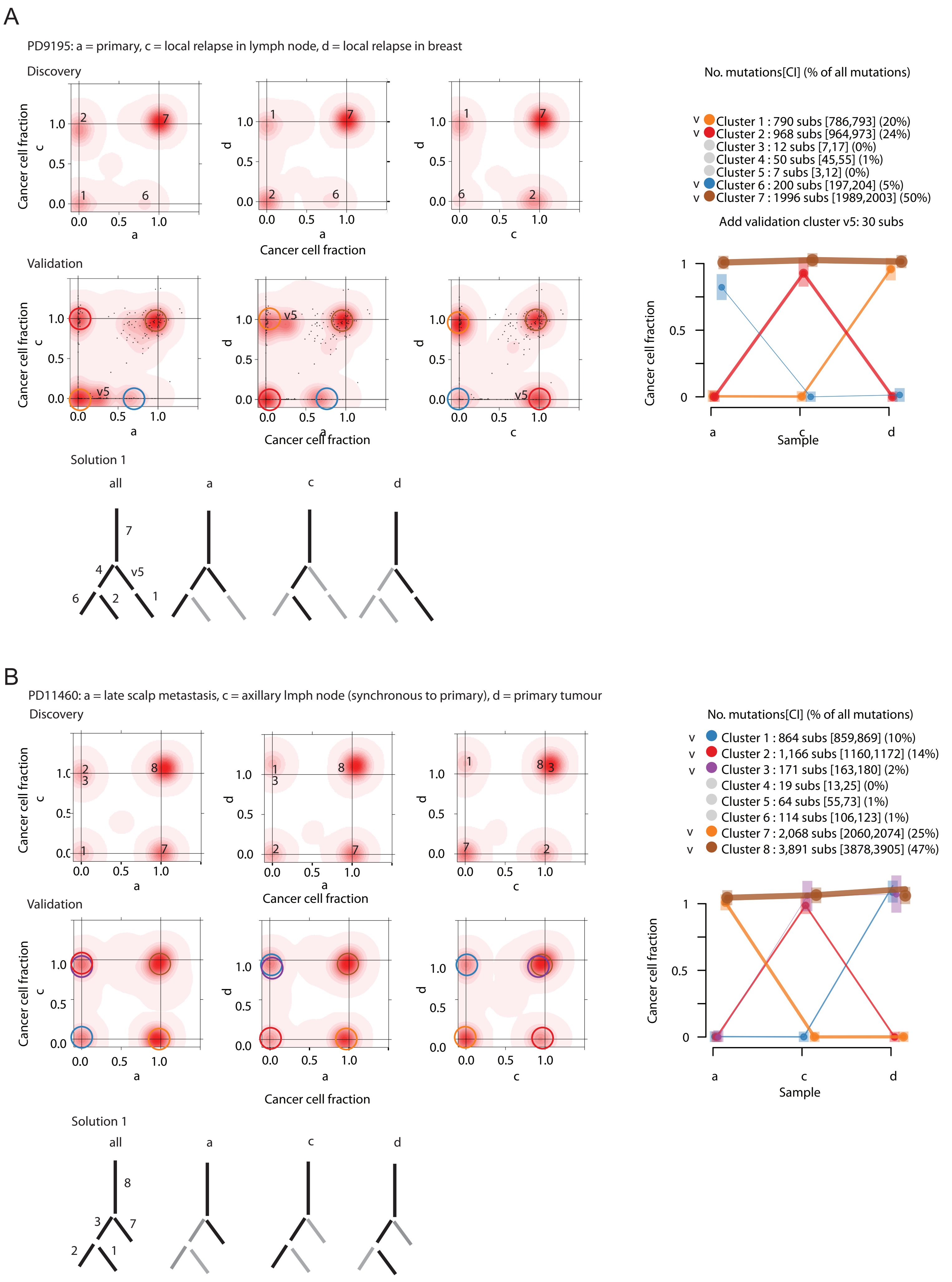
A
PD9195: a = primary, c = local relapse in lymph node, d = local relapse in breast
Discovery
Validation
Cancer cell fraction
Solution 1
all
a
c
d
No. mutations[CI] (% of all mutations)
v Cluster 1 : 790 subs [786,793] (20%)
v Cluster 2 : 968 subs [964,973] (24%)
Cluster 3 : 12 subs [7,17] (0%)
Cluster 4 : 50 subs [45,55] (1%)
Cluster 5 : 7 subs [3,12] (0%)
v Cluster 6 : 200 subs [197,204] (5%)
v Cluster 7 : 1996 subs [1989,2003] (50%)
Add validation cluster v5: 30 subs
Sample
B
PD11460: a = late scalp metastasis, c = axillary lmph node (synchronous to primary), d = primary tumour
Discovery
Validation
Cancer cell fraction
Solution 1
No. mutations[CI] (% of all mutations)
v Cluster 1 : 864 subs [859,869] (10%)
v Cluster 2 : 1,166 subs [1160,1172] (14%)
v Cluster 3 : 171 subs [163,180] (2%)
Cluster 4 : 19 subs [13,25] (0%)
Cluster 5 : 64 subs [55,73] (1%)
Cluster 6 : 114 subs [106,123] (1%)
v Cluster 7 : 2,068 subs [2060,2074] (25%)
v Cluster 8 : 3,891 subs [3878,3905] (47%)
Sample

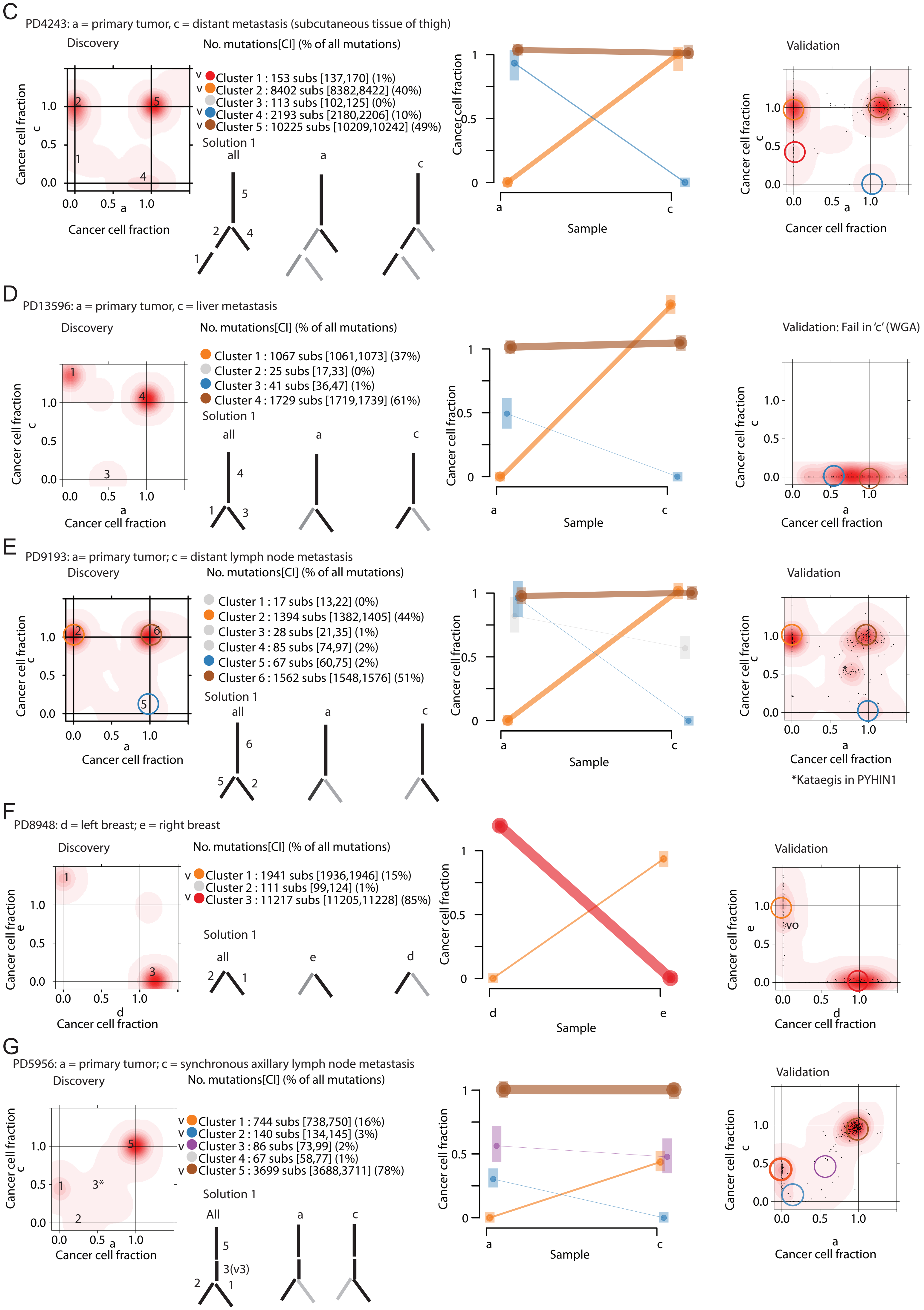

C
PD4243: a = primary tumor, c = distant metastasis (subcutaneous tissue of thigh)
Discovery
No. mutations[CI] (% of all mutations)
v Cluster 1 : 153 subs [137,170] (1%)
v Cluster 2 : 8402 subs [8382,8422] (40%)
Cluster 3 : 113 subs [102,125] (0%)
v Cluster 4 : 2193 subs [2180,2206] (10%)
v Cluster 5 : 10225 subs [10209,10242] (49%)
Solution 1
all
a
c
Cancer cell fraction
Sample
Validation
D
PD13596: a = primary tumor, c = liver metastasis
Discovery
No. mutations[CI] (% of all mutations)
Cluster 1 : 1067 subs [1061,1073] (37%)
Cluster 2 : 25 subs [17,33] (0%)
Cluster 3 : 41 subs [36,47] (1%)
Cluster 4 : 1729 subs [1719,1739] (61%)
Solution 1
Validation: Fail in 'c' (WGA)
E
PD9193: a= primary tumor; c = distant lymph node metastasis
Discovery
No. mutations[CI] (% of all mutations)
Cluster 1 : 17 subs [13,22] (0%)
Cluster 2 : 1394 subs [1382,1405] (44%)
Cluster 3 : 28 subs [21,35] (1%)
Cluster 4 : 85 subs [74,97] (2%)
Cluster 5 : 67 subs [60,75] (2%)
Cluster 6 : 1562 subs [1548,1576] (51%)
Solution 1
Validation
*Kataegis in PYHIN1
F
PD8948: d = left breast; e = right breast
Discovery
No. mutations[CI] (% of all mutations)
v Cluster 1 : 1941 subs [1936,1946] (15%)
Cluster 2 : 111 subs [99,124] (1%)
v Cluster 3 : 11217 subs [11205,11228] (85%)
Solution 1
all
e
d
Validation
vo
G
PD5956: a = primary tumor; c = synchronous axillary lymph node metastasis
Discovery
No. mutations[CI] (% of all mutations)
v Cluster 1 : 744 subs [738,750] (16%)
v Cluster 2 : 140 subs [134,145] (3%)
v Cluster 3 : 86 subs [73,99] (2%)
Cluster 4 : 67 subs [58,77] (1%)
v Cluster 5 : 3699 subs [3688,3711] (78%)
Solution 1
All
3(v3)
3*
Validation

H

PD9194: a = primary tumour, c = local relapse (breast).

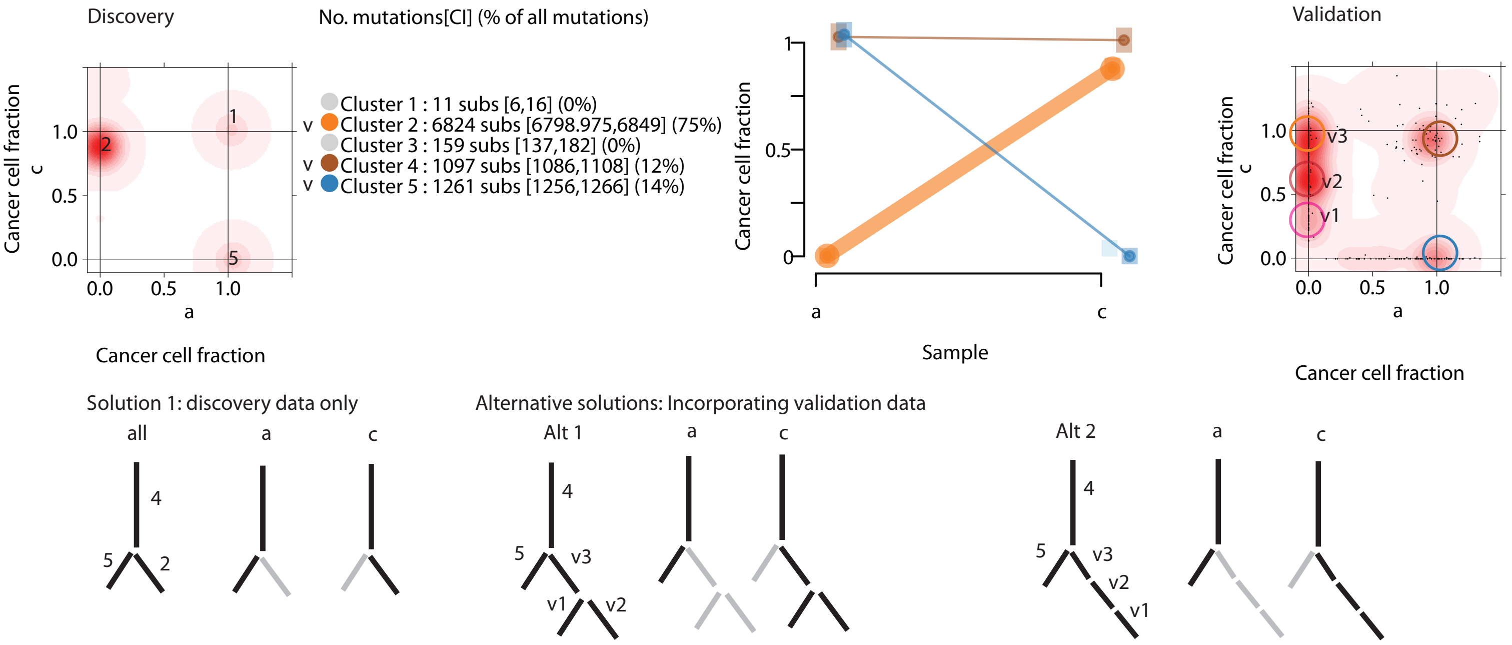

I

PD4252: a = primary tumor; c = synchronous lymph node metastasis

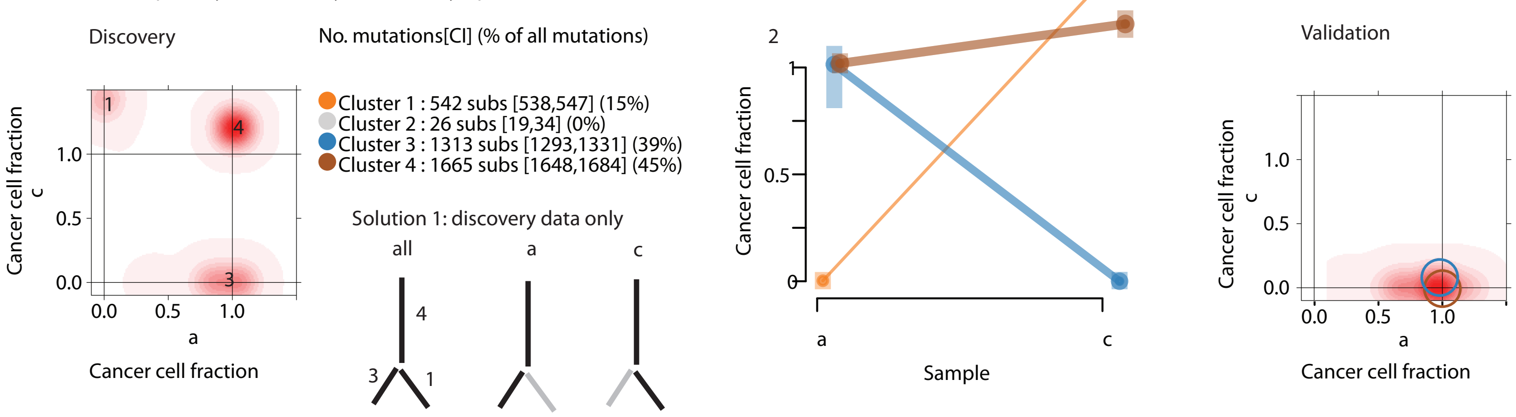

J

PD114780: a,d = primary tumor (2 separate foci in muti-focal cancer); e = synchronous axillary lymph node metastasis

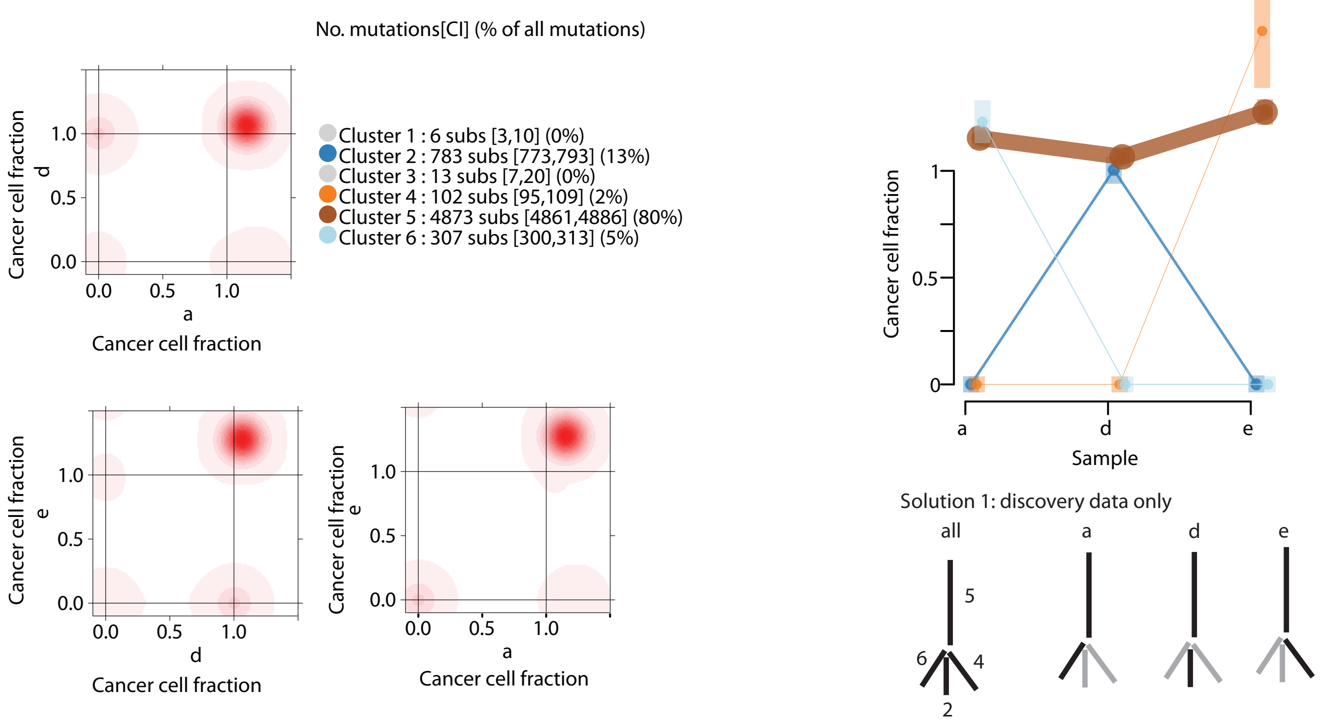

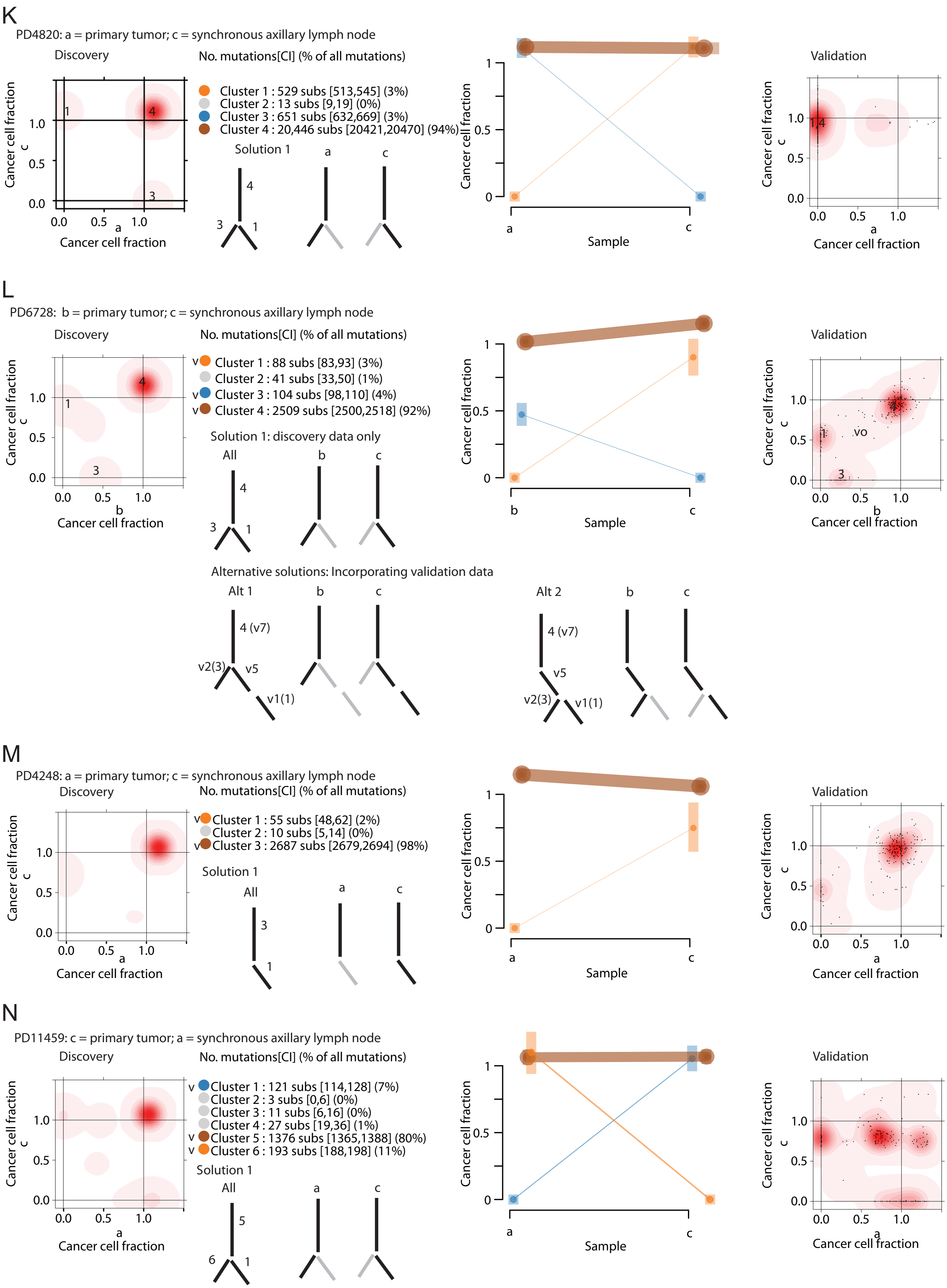
K
PD4820: a = primary tumor; c = synchronous axillary lymph node
Discovery
No. mutations[CI] (% of all mutations)
Cluster 1 : 529 subs [513,545] (3%)
Cluster 2 : 13 subs [9,19] (0%)
Cluster 3 : 651 subs [632,669] (3%)
Cluster 4 : 20,446 subs [20421,20470] (94%)
Solution 1
Cancer cell fraction
Sample
Validation
L
PD6728: b = primary tumor; c = synchronous axillary lymph node
Discovery
No. mutations[CI] (% of all mutations)
v Cluster 1 : 88 subs [83,93] (3%)
Cluster 2 : 41 subs [33,50] (1%)
v Cluster 3 : 104 subs [98,110] (4%)
v Cluster 4 : 2509 subs [2500,2518] (92%)
Solution 1: discovery data only
All
Alternative solutions: Incorporating validation data
Alt 1
4 (v7)
v2(3)
v5
v1(1)
Alt 2
Cancer cell fraction
Sample
Validation
M
PD4248: a = primary tumor; c = synchronous axillary lymph node
Discovery
No. mutations[CI] (% of all mutations)
v Cluster 1 : 55 subs [48,62] (2%)
Cluster 2 : 10 subs [5,14] (0%)
v Cluster 3 : 2687 subs [2679,2694] (98%)
Solution 1
All
Cancer cell fraction
Sample
Validation
N
PD11459: c = primary tumor; a = synchronous axillary lymph node
Discovery
No. mutations[CI] (% of all mutations)
v Cluster 1 : 121 subs [114,128] (7%)
Cluster 2 : 3 subs [0,6] (0%)
Cluster 3 : 11 subs [6,16] (0%)
Cluster 4 : 27 subs [19,36] (1%)
v Cluster 5 : 1376 subs [1365,1388] (80%)
v Cluster 6 : 193 subs [188,198] (11%)
Solution 1
All
Cancer cell fraction
Sample
Validation

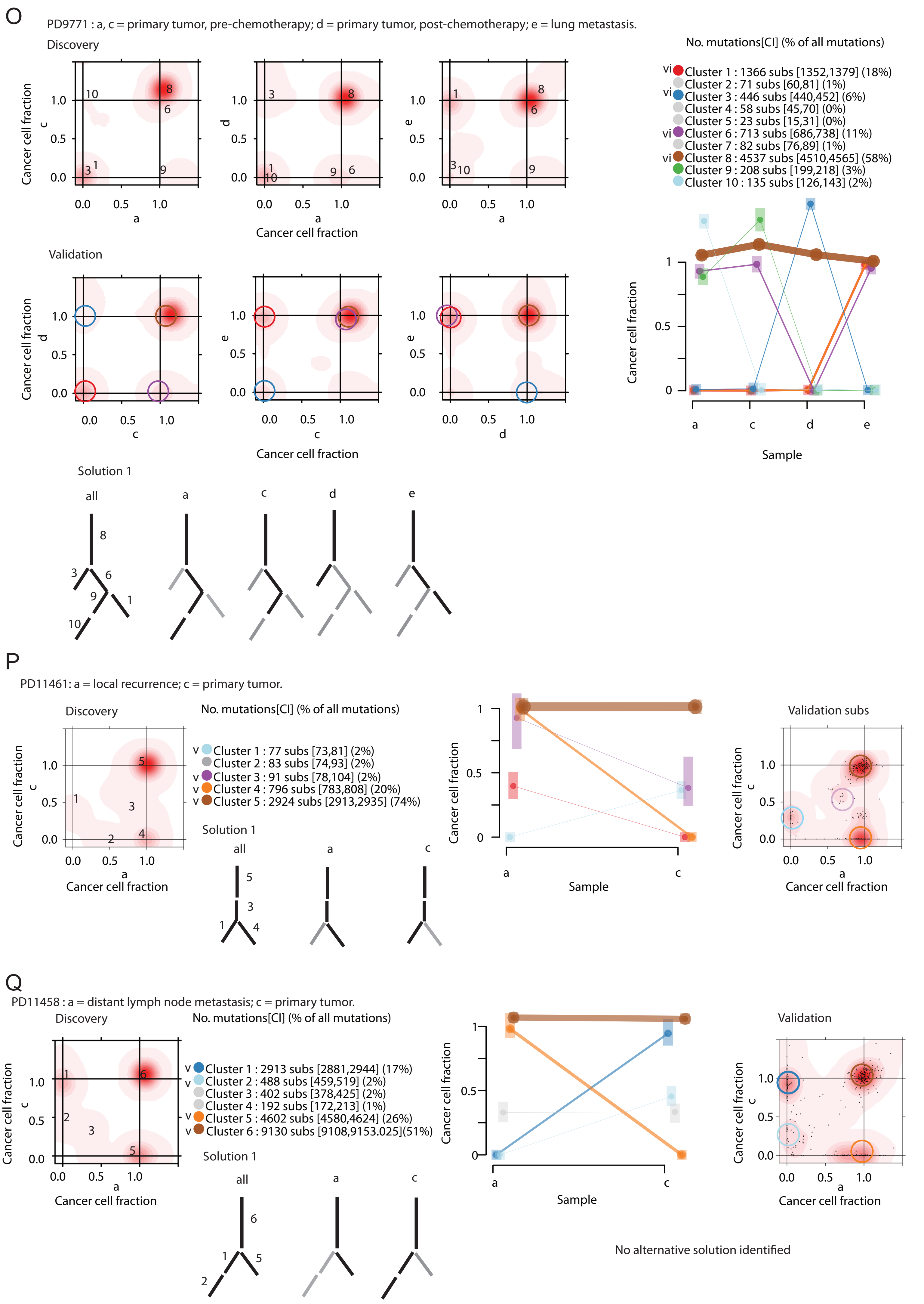
O
PD9771 : a, c = primary tumor, pre-chemotherapy; d = primary tumor, post-chemotherapy; e = lung metastasis.
Discovery
Cancer cell fraction
Validation
No. mutations[CI] (% of all mutations)
vi Cluster 1 : 1366 subs [1352,1379] (18%)
Cluster 2 : 71 subs [60,81] (1%)
vi Cluster 3 : 446 subs [440,452] (6%)
Cluster 4 : 58 subs [45,70] (0%)
Cluster 5 : 23 subs [15,31] (0%)
vi Cluster 6 : 713 subs [686,738] (11%)
Cluster 7 : 82 subs [76,89] (1%)
vi Cluster 8 : 4537 subs [4510,4565] (58%)
Cluster 9 : 208 subs [199,218] (3%)
Cluster 10 : 135 subs [126,143] (2%)
Sample
Solution 1
all
a
c
d
e
P
PD11461: a = local recurrence; c = primary tumor.
Discovery
No. mutations[CI] (% of all mutations)
v Cluster 1 : 77 subs [73,81] (2%)
Cluster 2 : 83 subs [74,93] (2%)
v Cluster 3 : 91 subs [78,104] (2%)
v Cluster 4 : 796 subs [783,808] (20%)
v Cluster 5 : 2924 subs [2913,2935] (74%)
Solution 1
Validation subs
Cancer cell fraction
Sample
Q
PD11458 : a = distant lymph node metastasis; c = primary tumor.
Discovery
No. mutations[CI] (% of all mutations)
v Cluster 1 : 2913 subs [2881,2944] (17%)
v Cluster 2 : 488 subs [459,519] (2%)
Cluster 3 : 402 subs [378,425] (2%)
Cluster 4 : 192 subs [172,213] (1%)
v Cluster 5 : 4602 subs [4580,4624] (26%)
v Cluster 6 : 9130 subs [9108,9153.025](51%)
Solution 1
Validation
Cancer cell fraction
Sample
No alternative solution identified

Figure S1, related to Figure 1. Phylogenetic Trees Derived from Whole Genome Data Describe the Evolution of 17 Relapsed or Metastatic Breast Cancers.

(A–Q) Depiction of the approaches used to construct one of the 17 phylogenetic trees represented in Figure 1 using multi-sample genome-wide somatic substitution data. Each panel depicts to a single cancer and contains 3 elements including possible phylogenetic tree structures, mutation density plots and cancer cell fraction line plots. For each patient, density plots of cancer cell fractions were derived by applying a multi-dimensional Bayesian Dirichlet process to whole genome data (discovery) and independently to variants selected for inclusion in the high depth re-sequencing (validation) experiment (16/17 patients). The cancer cell fraction refers to the proportion of tumour cells within a sample, estimated to harbor that cluster of mutations. Within the discovery density plots each significant cluster (those containing 2% or greater of mutations) is annotated with a number that refers to the relevant mutation cluster as reported in the legend. The legend reports the number of mutations in each cluster and their 95% credible intervals (CI) and dictates the relative branch lengths of the phylogenetic trees reported in Figure 1. Across 13 cases, 45 out of 48 discovery clusters were independently identified through validation data clustering, demarcated as a ‘v’ in the legend and colored circles within the validation density plots. Additional clusters identified by the validation experiment, but not significant in the discovery experiment, are annotated by a ‘v’ within the density plot. For three individuals, validation pulldown failed on account of whole genome amplification (WGA) technical failure (D, I, K). Line-plots report the cancer cell fraction of each cluster in related samples and the 95% credible intervals are depicted as underlying translucent colored bars while the line thickness reflects the number of mutations. Tree structures are constructed by hierarchical ordering of mutation clusters following the ‘pigeon-hole principle’. All tree solutions that are compatible with these data are presented. In each case the discovery data was consistent with a single solution and in 4 cases (A, C, L, H) an alternative solution was identified using additional information from high depth re-sequencing (validation) data.

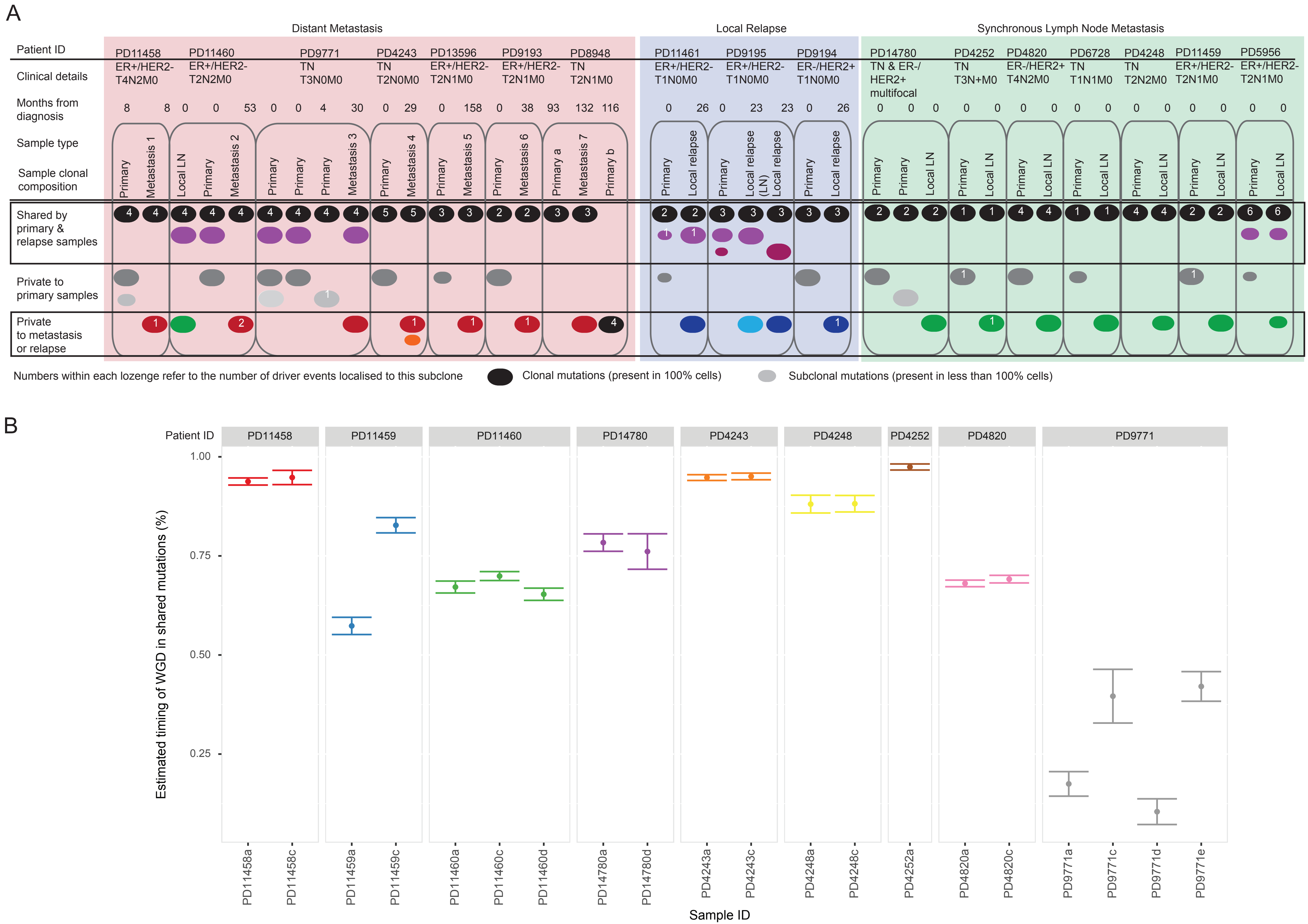
A
Distant Metastasis
Local Relapse
Synchronous Lymph Node Metastasis
Patient ID
Clinical details
Months from diagnosis
Sample type
Sample clonal composition
PD11458 ER+/HER2- T4N2M0
PD11460 ER+/HER2- T2N2M0
PD9771 TN T3N0M0
PD4243 TN T2N0M0
PD13596 ER+/HER2- T2N1M0
PD9193 ER+/HER2- T2N1M0
PD8948 TN T2N1M0
PD11461 ER+/HER2- T1N0M0
PD9195 ER+/HER2- T1N0M0
PD9194 ER-/HER2+ T1N0M0
PD14780 TN & ER-/ HER2+ multifocal
PD4252 TN T3N+M0
PD4820 ER-/HER2+ T4N2M0
PD6728 TN T1N1M0
PD4248 TN T2N2M0
PD11459 ER+/HER2- T2N1M0
PD5956 ER+/HER2- T2N1M0
8 8 0 0 53 0 0 4 30 0 29 0 158 0 38 93 132 116
0 26 0 23 23 0 26
0 0 0 0 0 0 0 0 0 0 0 0 0 0 0
Primary
Metastasis 1
Local LN
Primary
Metastasis 2
Primary
Primary
Primary
Metastasis 3
Primary
Metastasis 4
Primary
Metastasis 5
Primary
Metastasis 6
Primary a
Metastasis 7
Primary b
Primary
Local relapse
Primary
Local relapse (LN)
Local relapse
Primary
Local relapse
Primary
Primary
Local LN
Primary
Local LN
Primary
Local LN
Primary
Local LN
Primary
Local LN
Primary
Local LN
Primary
Local LN
Shared by primary & relapse samples
Private to primary samples
Private to metastasis or relapse
Numbers within each lozenge refer to the number of driver events localised to this subclone
Clonal mutations (present in 100% cells)
Subclonal mutations (present in less than 100% cells)
B
Patient ID
PD11458
PD11459
PD11460
PD14780
PD4243
PD4248
PD4252
PD4820
PD9771
Estimated timing of WGD in shared mutations (%)
1.00
0.75
0.50
0.25
PD11458a
PD11458c
PD11459a
PD11459c
PD11460a
PD11460c
PD11460d
PD14780a
PD14780d
PD4243a
PD4243c
PD4248a
PD4248c
PD4252a
PD4820a
PD4820c
PD9771a
PD9771c
PD9771d
PD9771e
Sample ID

Figure S2, Related to Figure 1. Subclonal Structure and Whole-Genome Duplication Timing During the Evolution of 17 Breast Cancers.

(A) Subclonal composition of 40 primary and relapse tumour samples from 17 patients were inferred from nd-Dirichlet clustering of genome-wide somatic substitution data. The subclonal composition of each tumour sample is presented within a single column and reveals that subclones can be present in different proportions in different samples from the same cancer. The proportion of each lozenge blocked with color reflects the proportion of cells in that sample that contain the mutations that constitute the same color branch of the relevant phylogenetic tree in Figure 1. Black lozenges represent clonal mutations, i.e. those present in 100% of cancer cells. The number of identified driver events within each subclone (branch) is reported in the relevant lozenge.

(B) Whole genome duplication timing estimated from genome-wide somatic substitution data from 20 tumour samples from 9 breast cancers where a whole genome duplication event had occurred. Dots correspond to the observed values and the error bars were generated through bootstrapping estimates of the number of observed mutations. The duplication estimate reflects the point in molecular time, within the phylogenetic tree trunk that the duplication event occurred. For each cancer duplication precedes primary-relapse clone divergence. Sample PD8948 is excluded due to technical limitations as discussed in Figure S3.

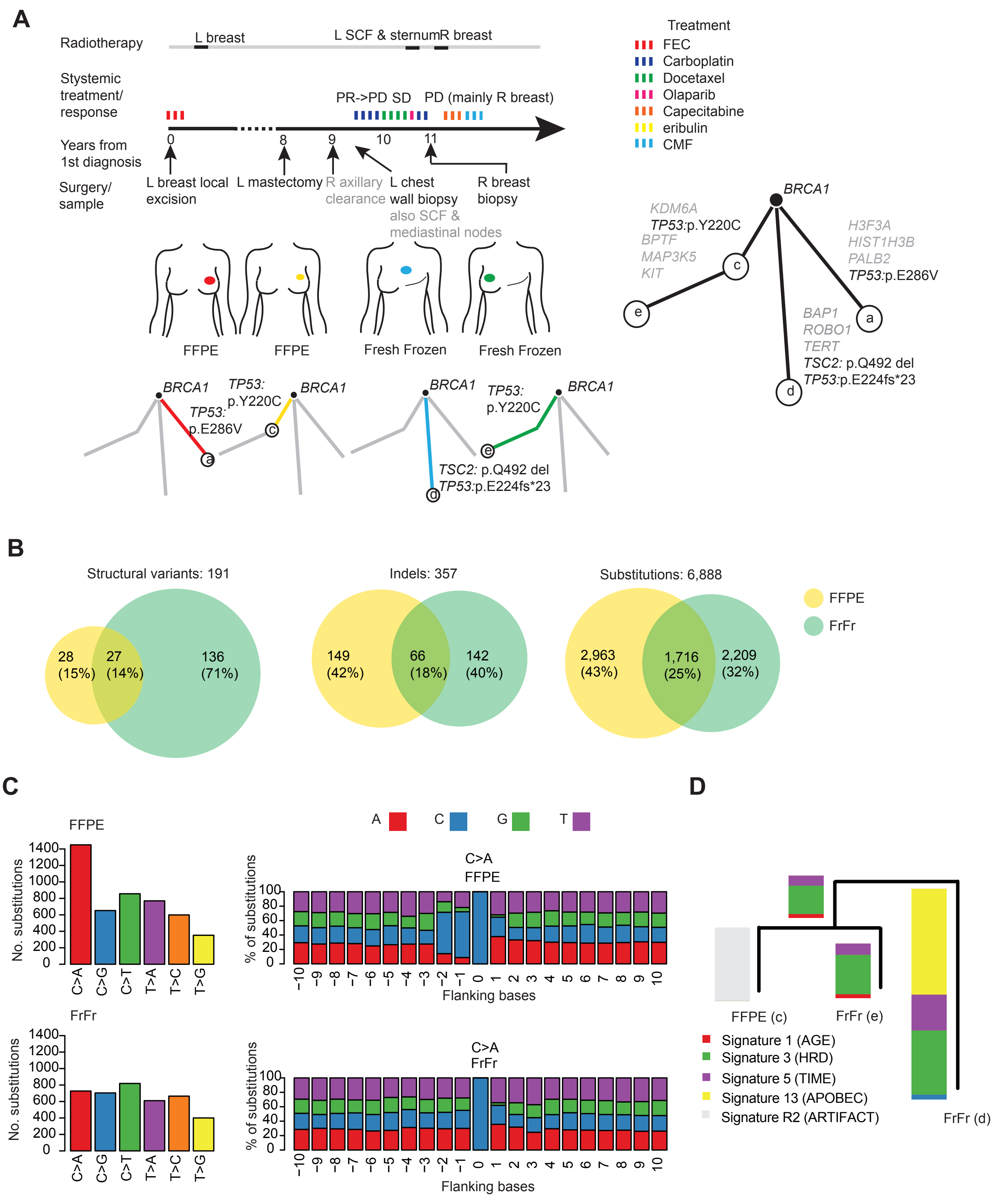
A
Radiotherapy
L breast
L SCF & sternum
R breast
Stystemic treatment/ response
PR->PD SD
PD (mainly R breast)
Years from 1st diagnosis
0
8
9
10
11
Surgery/ sample
L breast local excision
L mastectomy
R axillary clearance
L chest wall biopsy also SCF & mediastinal nodes
R breast biopsy
Treatment
FEC
Carboplatin
Docetaxel
Olaparib
Capecitabine
eribulin
CMF
FFPE
FFPE
Fresh Frozen
Fresh Frozen
BRCA1
TP53: p.E286V
TP53: p.Y220C
BRCA1
BRCA1
TP53: p.Y220C
BRCA1
TSC2: p.Q492 del
TP53:p.E224fs*23
BRCA1
KDM6A
TP53:p.Y220C
BPTF
MAP3K5
KIT
H3F3A
HIST1H3B
PALB2
TP53:p.E286V
BAP1
ROBO1
TERT
TSC2: p.Q492 del
TP53:p.E224fs*23
B
Structural variants: 191
28 (15%)
27 (14%)
136 (71%)
Indels: 357
149 (42%)
66 (18%)
142 (40%)
Substitutions: 6,888
2,963 (43%)
1,716 (25%)
2,209 (32%)
FFPE
FrFr
C
FFPE
No. substitutions
C>A
C>G
C>T
T>A
T>C
T>G
FrFr
A
C
G
T
C>A FFPE
C>A FrFr
% of substitutions
Flanking bases
D
FFPE (c)
FrFr (e)
FrFr (d)
Signature 1 (AGE)
Signature 3 (HRD)
Signature 5 (TIME)
Signature 13 (APOBEC)
Signature R2 (ARTIFACT)

Figure S3, related to Figure 2. Genome-Wide Sequencing of Four Samples from a Single Patient Reveals Serial Unrelated Cancers.

(A) Analysis of genomic sequence data reveals that multiple breast tumour samples in a known *BRCA1* mutation carrier (case PD8948) were derived from three different cancers. The clinical history in relation to the acquisition of the four sequenced cancer samples is presented. The nature of each sample – either fresh frozen (FrFr) or formalin fixed paraffin embedded (FFPE) is specified. Treatments are annotated in relation to time and treatment responses reported (PR = partial response, PD = progressive disease, SD = stable disease). A mock phylogenetic tree structure was determined based on non-synonymous point mutations identified within the scope of the 365 gene targeted panel across all four of the sequenced samples. Mutated genes annotate relevant tree branches where black font indicates a driver mutation. For each sample the phylogenetic tree branches (subclones) detected in that sample are highlighted in the same colour on the mock tree below. The tree derived from whole genome sequence data that was available for three of the samples (a, c and e) features in Figure 1.
(B-D) Genome-wide mutational analysis of two samples (PD8948c and PD8948e) thought to be clonally related based on targeted capture (A). One sample (PD8948c) is derived from formalin fixed paraffin embedded (FFPE) tissue and the other (PD8948e) from fresh frozen tissue (FrFr). (B) Venn diagrams demonstrate a significant overlap amongst all mutation types confirming that these samples are clonally related. As expected the sample obtained 3 years later (PD8948e) contains a significant private mutation burden. Unexpectedly, the earlier sample (PD8948c) contained a similar private mutation burden raising the suspicion of a sequencing artifact. (C) The substitution (base change) spectra in the two samples is presented (left), and demonstrates a predominance of C>A base changes. The flanking sequence 10 base pairs either side of each C>A mutation in the FFPE and related fresh frozen samples are presented (right) and show an enrichment of C at the -1 and -2 genomic positions. (D) Phylogenetic tree construction of this sample and formal mutational signature analysis using a non-negative matrix factorization approach assigned all mutations private to the FFPE sample as deriving from a likely sequencing artifact similar to that previously reported as arising as a consequence of oxidation during exome library preparation. Mutation signatures shared by the two samples were consistent with those private to the relapse (FrFr) sample being dominated by a signature of homologous recombination deficiency (HRD) as expected in this patient with a germline *BRCA1* mutation.

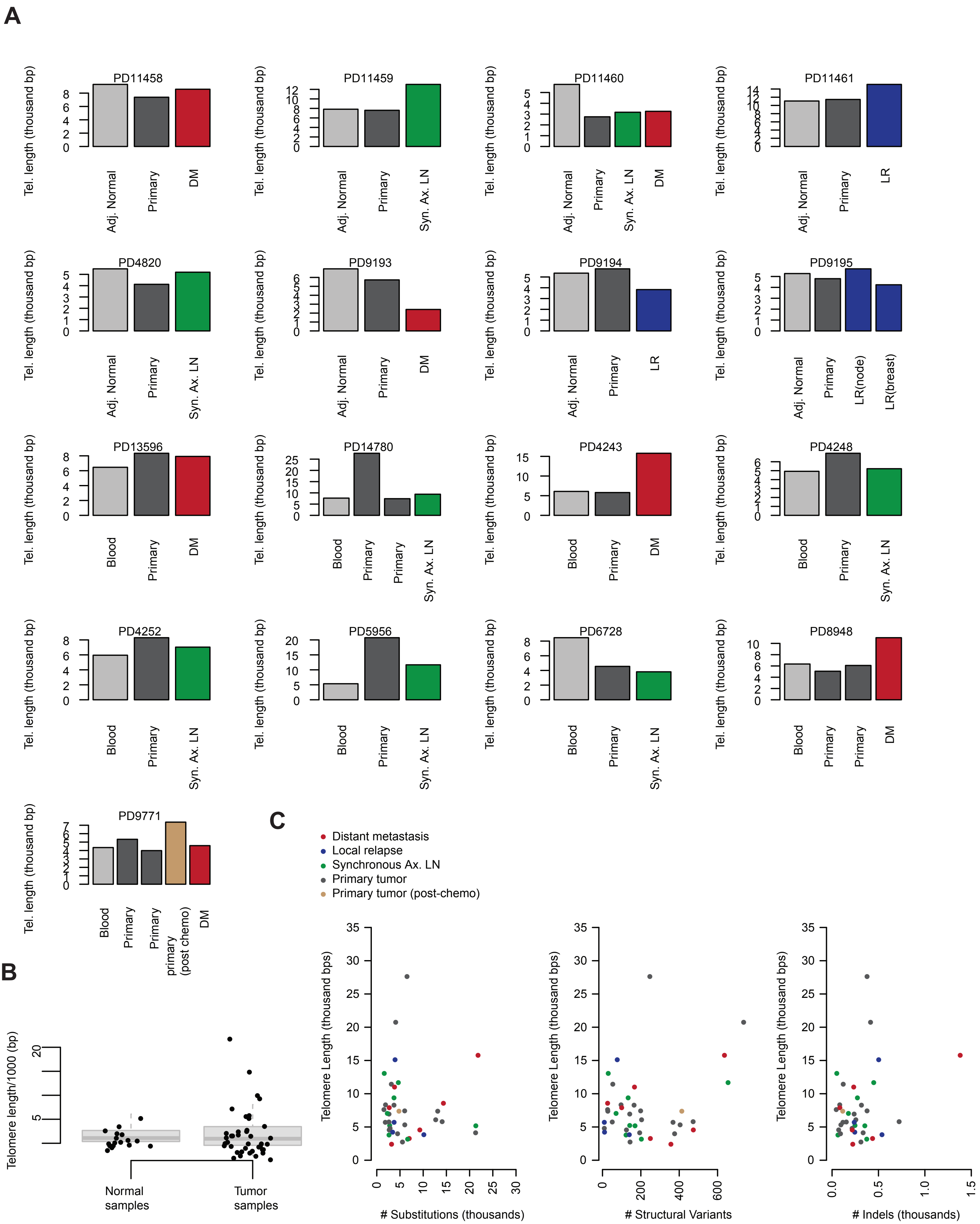

A
PD11458
PD11459
PD11460
PD11461
PD4820
PD9193
PD9194
PD9195
PD13596
PD14780
PD4243
PD4248
PD4252
PD5956
PD6728
PD8948
PD9771
Tel. length (thousand bp)
Adj. Normal
Primary
DM
Syn. Ax. LN
LR
LR(node)
LR(breast)
Blood
primary (post chemo)
B
Telomere length/1000 (bp)
Normal samples
Tumor samples
C
Distant metastasis
Local relapse
Synchronous Ax. LN
Primary tumor
Primary tumor (post-chemo)
Telomere Length (thousand bps)
Substitutions (thousands)
Structural Variants
Indels (thousands)

Figure S4, related to Figure 3. Telomere Lengths of 57 Breast Cancer and Matched Normal Samples from 17 Individuals.

(A) Barplots indicate telomere lengths estimated for all sequenced samples from an individual and include either a blood or adjacent normal breast tissue (Adj. Normal) derived germline sample (light grey bars), in addition to tumour samples from primary tumour(s) (dark grey bars) and relapsed or metastatic sample(s) where LR = local relapse (blue bars), DM = distant metastasis (red bars), Syn. Ax. LN = synchronous axillary lymph node samples (green bars).
(B) Boxplots of telomere lengths in normal and tumour samples where the box represents the inter-quartile range (IQR) dissected by the median, whiskers represent the maximum and minimum range of the data that does not exceed 1.5x the IQR while any outlier data points extend beyond this. Bp = Base pairs.
(C) Scatterplots relate telomere lengths to the number of somatic substitutions within 39 tumour samples from 17 patients (excludes FFPE derived sample PD8948c as this estimate is thought to be inaccurate due to technical artifacts introduced by the fixation method).

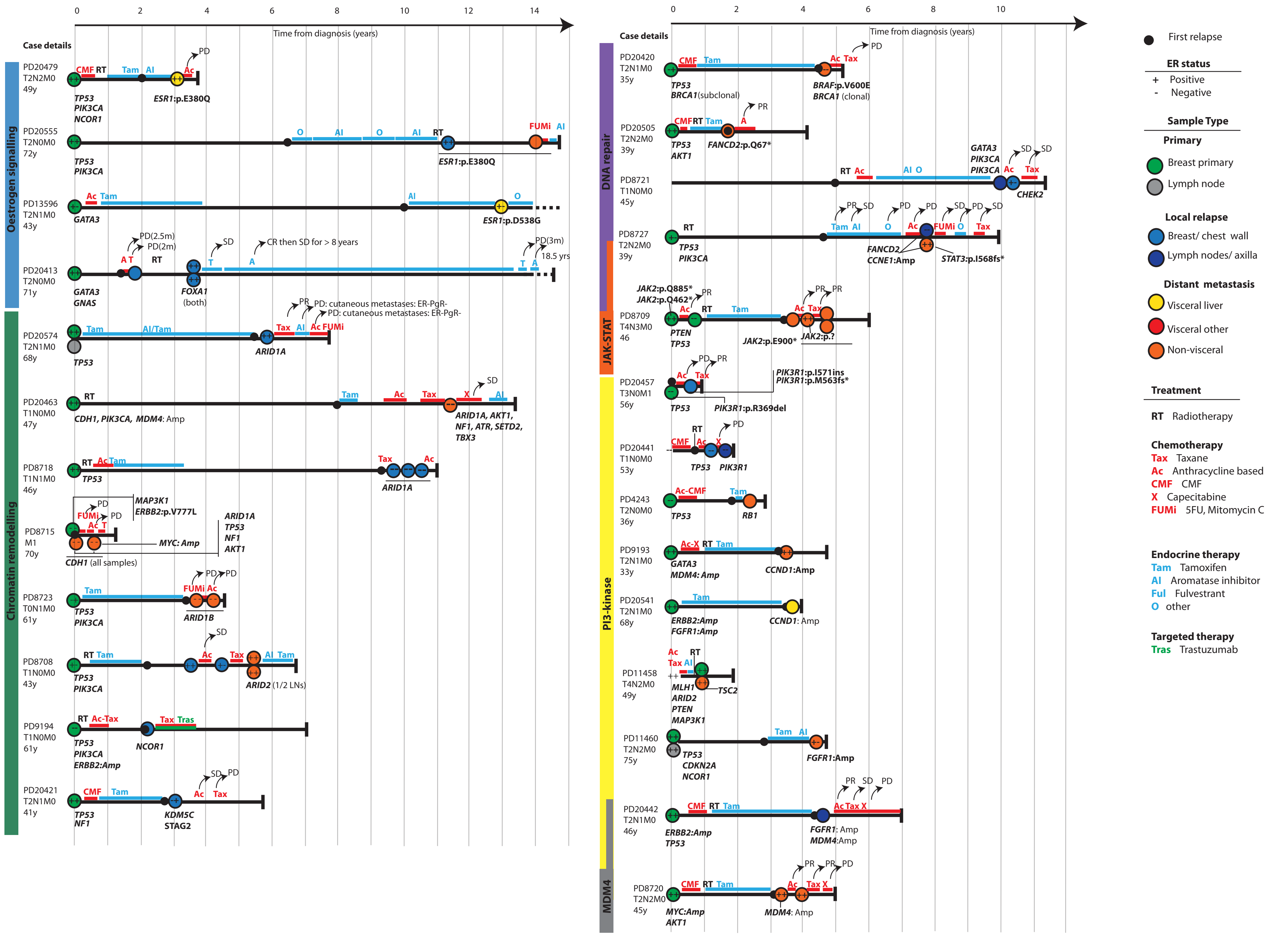

Time from diagnosis (years)
0
2
4
6
8
10
12
14
Case details
Oestrogen signalling
Chromatin remodelling
DNA repair
JAK-STAT
PI3-kinase
MDM4
PD20479
T2N2M0
49y
TP53
PIK3CA
NCOR1
ESR1:p.E380Q
PD20555
T2N0M0
72y
TP53
PIK3CA
ESR1:p.E380Q
PD13596
T2N1M0
43y
GATA3
ESR1:p.D538G
PD20413
T2N0M0
71y
GATA3
GNAS
FOXA1
(both)
CR then SD for > 8 years
18.5 yrs
PD20574
T2N1M0
68y
TP53
ARID1A
PD: cutaneous metastases: ER-PgR-
PD: cutaneous metastases: ER-PgR-
PD20463
T1N0M0
47y
CDH1, PIK3CA, MDM4: Amp
ARID1A, AKT1,
NF1, ATR, SETD2,
TBX3
PD8718
T1N1M0
46y
TP53
ARID1A
PD8715
M1
70y
MAP3K1
ERBB2:p.V777L
MYC: Amp
ARID1A
TP53
NF1
AKT1
CDH1 (all samples)
PD8723
T0N1M0
61y
TP53
PIK3CA
ARID1B
PD8708
T1N0M0
43y
TP53
PIK3CA
ARID2 (1/2 LNs)
PD9194
T1N0M0
61y
TP53
PIK3CA
ERBB2:Amp
NCOR1
PD20421
T2N1M0
41y
TP53
NF1
KDM5C
STAG2
PD20420
T2N1M0
35y
TP53
BRCA1(subclonal)
BRAF:p.V600E
BRCA1 (clonal)
PD20505
T2N2M0
39y
TP53
AKT1
FANCD2:p.Q67*
PD8721
T1N0M0
45y
GATA3
PIK3CA
PIK3CA
CHEK2
PD8727
T2N2M0
39y
TP53
PIK3CA
FANCD2
CCNE1:Amp
STAT3:p.I568fs*
JAK2:p.Q885*
JAK2:p.Q462*
PD8709
T4N3M0
46
PTEN
TP53
JAK2:p.E900*
JAK2:p.?
PD20457
T3N0M1
56y
TP53
PIK3R1:p.R369del
PIK3R1:p.I571ins
PIK3R1:p.M563fs*
PD20441
T1N0M0
53y
TP53
PIK3R1
PD4243
T2N0M0
36y
TP53
RB1
PD9193
T2N1M0
33y
GATA3
MDM4: Amp
CCND1:Amp
PD20541
T2N1M0
68y
ERBB2:Amp
FGFR1:Amp
CCND1: Amp
PD11458
T4N2M0
49y
MLH1
ARID2
PTEN
MAP3K1
TSC2
PD11460
T2N2M0
75y
TP53
CDKN2A
NCOR1
FGFR1:Amp
PD20442
T2N1M0
46y
ERBB2:Amp
TP53
FGFR1: Amp
MDM4:Amp
PD8720
T2N2M0
45y
MYC:Amp
AKT1
MDM4: Amp
First relapse
ER status
+ Positive
- Negative
Sample Type
Primary
Breast primary
Lymph node
Local relapse
Breast/ chest wall
Lymph nodes/ axilla
Distant metastasis
Visceral liver
Visceral other
Non-visceral
Treatment
RT Radiotherapy
Chemotherapy
Tax Taxane
Ac Anthracycline based
CMF CMF
X Capecitabine
FUMi 5FU, Mitomycin C
Endocrine therapy
Tam Tamoxifen
AI Aromatase inhibitor
Ful Fulvestrant
O other
Targeted therapy
Tras Trastuzumab

Figure S5, related to Figure 6. Driver Alterations Arising Late in the Evolution of 26 Relapsed Breast Cancers.

Clinical histories presented along a time line for 26 patients where a relapse specific driver mutation was identified within the scope of the 365 cancer gene panel. Patient ID, TNM stage and age at diagnosis are reported (left side). The extent of the black line, terminating in a vertical bar, indicates time from diagnosis to death. The black circle reflects first diagnosis of relapse. Each colored circle represents a sequenced sample. Driver mutations are annotated according to where they first appear in chronological time in relation to samples sequenced. Where available, individual sample estrogen and progesterone receptor status (respectively) are reported within relevant circles with variation between individual samples identified in 9 cases. Chronological treatment exposures are indicated by colored horizontal lines and annotating text. Treatment response is annotated where known (PR = partial response, PD = progressive disease, SD = stable disease, CR = complete response).

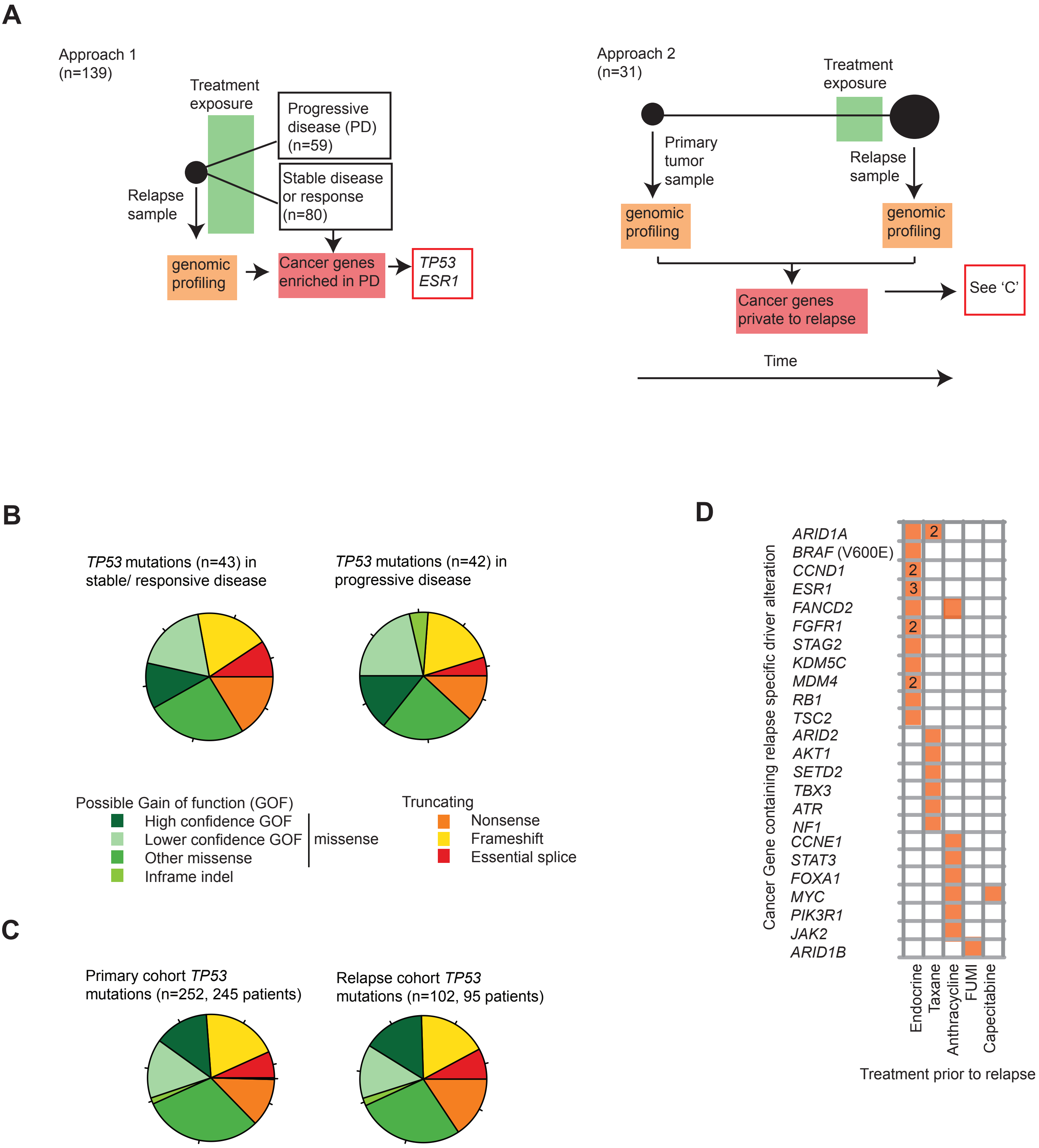
A
Approach 1
(n=139)
Treatment exposure
Progressive disease (PD) (n=59)
Stable disease or response (n=80)
Relapse sample
genomic profiling
Cancer genes enriched in PD
TP53
ESR1
Approach 2
(n=31)
Treatment exposure
Primary tumor sample
Relapse sample
genomic profiling
genomic profiling
Cancer genes private to relapse
See 'C'
Time
B
TP53 mutations (n=43) in stable/ responsive disease
TP53 mutations (n=42) in progressive disease
Possible Gain of function (GOF)
High confidence GOF
Lower confidence GOF
Other missense
Inframe indel
missense
Truncating
Nonsense
Frameshift
Essential splice
C
Primary cohort TP53 mutations (n=252, 245 patients)
Relapse cohort TP53 mutations (n=102, 95 patients)
D
Cancer Gene containing relapse specific driver alteration
ARID1A
BRAF (V600E)
CCND1
ESR1
FANCD2
FGFR1
STAG2
KDM5C
MDM4
RB1
TSC2
ARID2
AKT1
SETD2
TBX3
ATR
NF1
CCNE1
STAT3
FOXA1
MYC
PIK3R1
JAK2
ARID1B
2
2
3
2
2
Endocrine
Taxane
Anthracycline
FUMI
Capecitabine
Treatment prior to relapse

Figure S6, related to Figure 6. Treatment Exposures in Relation to Breast Cancer Evolution.

(A) Two approaches were used in the current study to identify potential associations between cancer genotype and treatment response. A total of 139 cases were identified that fulfilled the three criteria needed to perform ‘Approach 1’ that was designed to identify cancer genes that may be associated with disease progression through treatment. These criteria were: a) A sample from a relapsed breast cancer was obtained and sequenced, b) A documented treatment was administered shortly after this, and c) The best clinical response to the treatment was documented. *TP53* and *ESR1* were more frequently mutated in cancers that progressed rather than responded or stabilized after treatment (63% vs 45%, p = 0.04 and 7% vs 0%, p= 0.03 respectively, Fisher’s exact test). These trends were observed for both genes on subgroup analysis of chemotherapy and endocrine therapy but statistical significance was not reached due to small sample sizes. A total of 31 cases permitted ‘Approach 2’ that was designed to identify driver mutations potentially arising de novo during a treatment exposure, i.e. those private to post-treatment samples. This approach required that a) Both primary tumor and a subsequent relapse site were sampled and sequenced and b) A documented systemic treatment intervention was performed immediately prior to the latter. Numbers in brackets refer to the number of sample-treatment-response cases.

(B) Distribution of *TP53* mutation types amongst cancers according to response to therapy. Gain of function mutations are taken from Petitjean et al., 2007. No enrichment for missense compared to nonsense mutations is seen amongst samples that progressed compared to those that did not (p=1.0, fisher’s exact test). Numbers refer to the number of mutations.

(C) Distribution of *TP53* mutation types within the primary tumour (The Cancer Genome Atlas, TCGA) and relapse cohorts. Numbers refer to the number of mutations. *TP53* mutations were seen in 245/705 (35%) patients and 95/170 (56%) of patients in the primary and relapse cohorts respectively.

(D) Driver mutations private to post-treatment samples after exposure to a range of treatments. In 24/31 cases at least 1 new driver alteration (total = 33 alterations) not present in the primary tumour was detected in the relapse sample. Caution is needed in attributing mutations to specific exposures as these cancers were often exposed to multiple treatments prior to relapse.
